# Albumin-targeted oxaliplatin(iv) prodrugs bearing STING agonists†

**DOI:** 10.1039/d5qi00433k

**Published:** 2025-04-04

**Authors:** Martijn Dijkstra, Michael Gutmann, Mathias Gradl, Anja Federa, Carola Jaunecker, John Vasco Breitenstein, Petra Vician, Christine Pirker, Daniel Valcanover, Petra Heffeter, Bernhard K. Keppler, Walter Berger, Christian R. Kowol

**Affiliations:** a University of Vienna, Faculty of Chemistry, Institute of Inorganic Chemistry Waehringer Str. 42 1090 Vienna Austria christian.kowol@univie.ac.at; b University of Vienna, Vienna Doctoral School in Chemistry (DoSChem) Waehringer Str. 42 1090 Vienna Austria; c Center for Cancer Research and Comprehensive Cancer Center, Medical University of Vienna Borschkegasse 8a 1090 Vienna Austria walter.berger@meduniwien.ac.at; d Research Cluster “Translational Cancer Therapy Research” 1090 Vienna Austria

## Abstract

The anticancer platinum complex oxaliplatin exerts its activity through DNA damage and immune-stimulatory mechanisms, but is associated with adverse treatment side effects. Platinum(iv) complexes represent a promising prodrug strategy to improve tolerability and to enhance antitumor efficacy *via* attachment of additional bioactive ligands or tumor-targeting moieties. In the present study, oxaliplatin(iv) complexes containing immune-stimulatory STING agonists SR-717 or MSA-2 were synthesized and their biological properties were studied. Whereas the Pt-SR-717 compound was fast reduced, Pt-MSA-2 complexes displayed significantly higher reductive stability reflected by low *in vitro* cytotoxicity. Although the platinum(iv) complexes activated interferon regulatory factor (IRF) and NF-κB signaling pathways less effectively compared to the free STING agonists, reducing conditions elevated cytotoxicity and STING downstream signaling, particularly for MSA-2-containing prodrugs. Rapid albumin binding of a maleimide-containing Pt-MSA-2 derivative resulted in elevated plasma levels, prolonged blood circulation, and enhanced tumor accumulation of platinum in CT-26 tumor-bearing mice. The Pt-MSA-2 complexes triggered immune activation and cytokine secretion without hematotoxicity usually associated with free oxaliplatin. The albumin-targeted Pt-MSA-2 drug significantly inhibited tumor growth after intravenous application, while the non-maleimide complex was effective only when applied peritumorally. However, the effects were not enhanced compared to mono-treatment with oxaliplatin or MSA-2, indicating a lack of synergism between the two simultaneously released agents. Our results demonstrate that oxaliplatin(iv) complexes represent a valuable strategy for enhanced tumor-targeting and adverse effect reduction, but question the simultaneous release of STING agonists and free oxaliplatin as a potent strategy towards synergistic antineoplastic activity.

## Abstract 2

An oxaliplatin(iv)-maleimide prodrug containing the STING agonist MSA-2 was synthesized with improved pharmacological properties and diminished hematotoxicity. The complex exerted anticancer effects but did not outperform free oxaliplatin or MSA-2.

## Graphical abstract

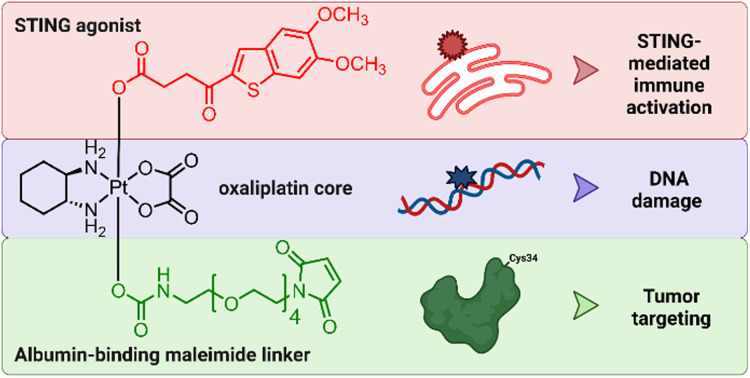

## Introduction

Since the breakthrough discovery of the anticancer activity of cisplatin, platinum(ii) complexes have prevailed as first-line treatment against a variety of cancer types, with approximately half of the present chemotherapies involving a platinum(ii) drug.^1^ Yet, patients suffer from serious side effects, such as systemic toxicity due to the lack of tumor specificity and the development of platinum resistance.^2,3^ In the past decades, platinum(iv) prodrugs were developed as a promising strategy to overcome the clinical limitations related to the use of platinum(ii) drugs.^4^ It is generally accepted that platinum(iv) complexes are much more inert, which limits premature deactivation by biomolecules. Additionally, platinum(iv) prodrugs release their cytotoxic platinum(ii) core *via* tumor-specific activation by reduction, either in the tumor microenvironment or inside the tumor cells after uptake *via* endocytosis,^5^ which reduces systemic toxicity.^6^ Although several platinum(iv) complexes, *e.g.* iproplatin and satraplatin, advanced into clinical trials, none have been approved so far.^7^ Over the past decades, research has increasingly focused on developing multi-action platinum(iv) complexes incorporating additional functional moieties, such as bioactive ligands or targeting groups. These modifications aim to enhance the antitumor efficacy of platinum(iv) drugs by synergistic effects or improving pharmacokinetics.^8^ In particular, human serum albumin (HSA) attracted significant attention as a versatile carrier for anticancer therapeutics, resulting in prolonged plasma half-lives of HSA–drug conjugates and increased intratumoral accumulation due to the enhanced permeability and retention (EPR) effect.^9^ Notable examples include the methotrexate–albumin conjugate MTX-HSA, which advanced into phase II clinical trials in combination therapy with cisplatin against advanced bladder cancer.^10^ Furthermore, aldoxorubicin, a maleimide-functionalized derivative of doxorubicin received orphan drug designation for the treatment of soft tissue sarcomas.^11^ Our group recently reported that maleimide functionalization of oxaliplatin(iv) complexes distinctly improved anticancer activity as compared to oxaliplatin *via* passive albumin targeting.^5,12–14^

In the last few decades, cancer immunotherapy reconstituted the field of oncology, manifesting plentiful FDA approvals of diverse novel immunotherapeutics, which is comprehensively reviewed elsewhere.^15^ There is compelling evidence that oxaliplatin also possesses immunological effects. Specifically, it is capable of inducing immunogenic cell death (ICD), a form of regulated cell death *via* activation of innate and adaptive immune responses, potentially resulting in potent and long-term antitumor effects.^16,17^ Accordingly, improving the anticancer activity of oxaliplatin by combinatorial treatment with immunomodulators attracted significant research interest lately. For example, a phase III trial evaluated oxaliplatin in combination with the PD-1 checkpoint inhibitor nivolumab as first-line treatment in patients with advanced gastric cancer, showing superior response rates as compared to cisplatin.^18^ The cyclic GMP-AMP synthase stimulator of interferon genes (cGAS-STING) pathway plays a crucial role in the activation of innate immunity and is therefore a popular target in modern drug discovery.^19^ Stimulation of the STING signaling pathway by the endogenous activator 2′,3′-cyclic guanosine monophosphate–adenosine monophosphate (cGAMP) induces the expression of type I interferons (IFNs) and proinflammatory cytokines, ultimately resulting in the activation of cytotoxic CD8^+^ T-cells. Activation of STING can be exploited therapeutically by small molecule agonists (*e.g.* cGAMP mimetics) to induce robust anticancer immune responses. Although diverse STING agonists advanced into clinical trials, as extensively reviewed by others,^20^ their clinical success is significantly challenged by several limiting factors, such as inadequate pharmacology, poor serum stability or high toxicity profiles due to ubiquitous STING expression, necessitating intratumoral administration for the majority of STING-targeting drugs.^21^ As a consequence, recent efforts focused on the development of delivery systems to improve the pharmacokinetic properties of STING agonists.^22^ For example, Mersana Therapeutics reported orphan drug designation of a novel HER2-targeting STING agonist antibody–drug conjugate for the treatment of patients with gastric cancer.^23^ In addition, several metal complexes have been studied for their ability to induce cGAS-STING pathway activation *via* DNA breakdown products, either from the nucleus or mitochondria.^24–27^ Recently, reports of two non-nucleotide cGAMP mimetics and STING agonists with promising antitumor activity, SR-717^28^ and MSA-2^29^, were published. SR-717 treatment led to superior tumor growth inhibition and enhanced overall survival compared to anti-PD-L1 therapy alone in a B16F10 melanoma mouse model. Moreover, MSA-2 induced regression of MC-38 syngeneic colorectal mouse tumors after oral administration and induced long-lasting anticancer immunity. Furthermore, in the case of MSA-2, numerous attempts have been reported to increase its clinical potential, *e.g.* by diverse structural modifications^30,31^ or by incorporation into platinum(ii) complexes,^32^ cisplatin(iv)-based prodrugs,^33^ or nanoparticles.^34,35^

Encouraged by our recent efforts in the development of tumor-targeted, triple-action platinum(iv) prodrugs bearing immunomodulators, such as the indoleamine 2,3-dioxygenase inhibitor 1-methyl-d-tryptophan,^12^ and envisioned by combining STING agonists and platinum(iv)-based chemotherapy for achievement of a synergistic antitumor effect, we investigated the potential of oxaliplatin(iv) prodrugs bearing the STING agonists SR-717 and MSA-2. The new complexes were synthesized and investigated *in vitro* to assess the cytotoxic activity, intracellular accumulation, and STING-dependent activation of the interferon regulatory factor (IRF) and nuclear factor kappa B (NF-κB) pathways. Finally, an albumin-binding maleimide-bearing platinum(iv)-MSA-2 derivative was synthesized and the pharmacokinetics, *i.e.* platinum tissue distribution as well as the immune activation and anticancer activity *in vivo* was studied in comparison with the single agents oxaliplatin and MSA-2, using syngeneic mouse models of colorectal cancer.

## Results and discussion

### Synthesis, stability and reduction of platinum(iv) complexes

**Scheme 1:**
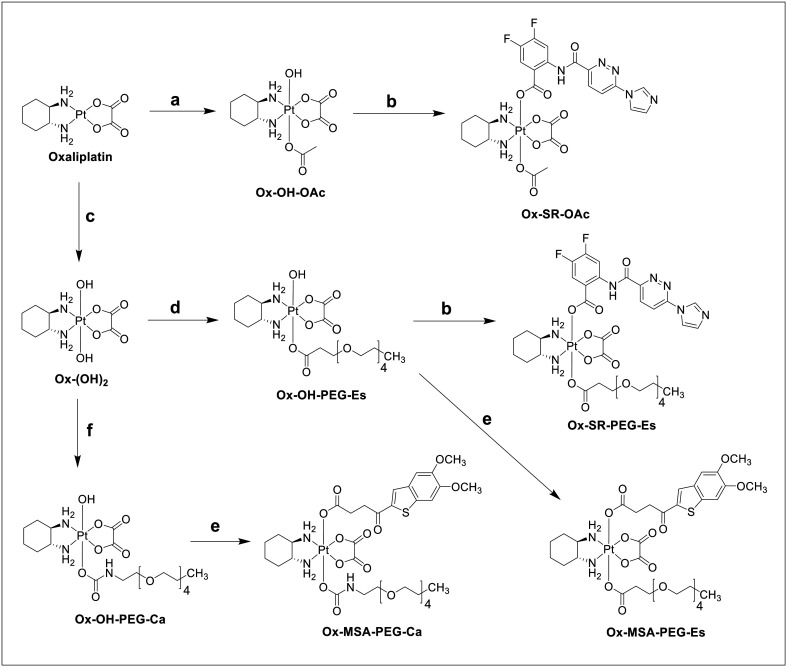
Synthetic routes to the final platinum(iv) complexes; (a) H_2_O_2_ (50 wt%) and acetic acid (AcOH) at room temperature (RT); (b) SR-717, TBTU, TEA, and DMF at RT; (c) H_2_O_2_ (50 wt%) and MQ-H_2_O at RT; (d) PEG-isoBu anhydride (synthesis, see the Experimental section) and DMF at RT; (e) MSA-2, TBTU, TEA, and DMF at RT; (f) PEG-NH_2_ and DMF at RT.

To commence our study, we focused on the design of suitable platinum(iv) prodrugs based on the STING agonist SR-717. SR-717, Ox-OH-OAc and Ox-(OH)_2_ were prepared as described in the literature.^28,36^ SR-717 was activated with 2-(1*H*-benzotriazole-1-yl)-1,1,3,3-tetramethylaminium tetrafluoroborate (TBTU) and coupled to Ox-OH-OAc *via* an ester linkage to form Ox-SR-OAc (Scheme 1).

**Fig. 1:**
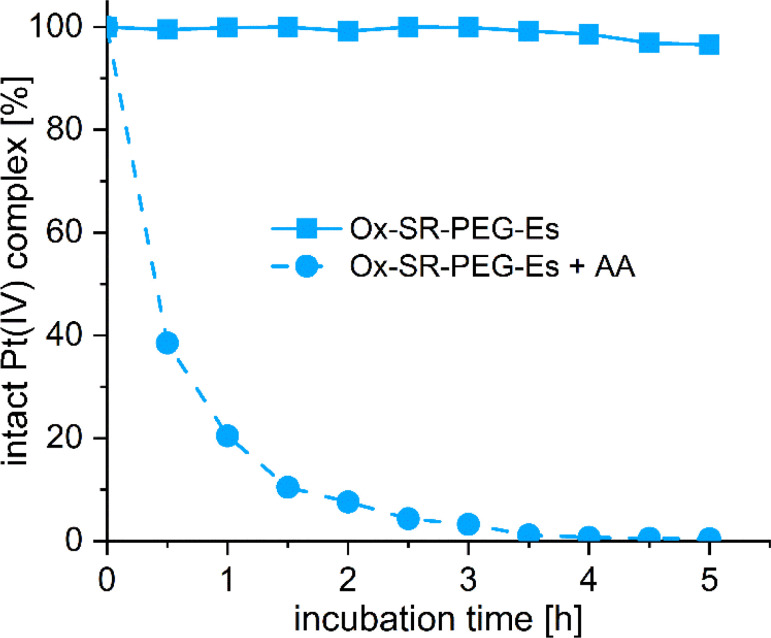
Stability and reduction kinetics of 1 mM Ox-SR-PEG-Es in 150 mM PB (pH = 7.4) at 20 °C without or with 10 eq. of AA over 5 h, measured with UHPLC.

Unfortunately, the aqueous solubility of Ox-SR-OAc was insufficient in various physiologically relevant media for further *in vivo* studies. We then shifted our strategy to Ox-SR-717 complexes bearing polyethylene glycol (PEG) linkers to increase solubility. 2,5,8,11-Tetraoxatetradecan-14-oic acid (PEG-COOH) was reacted with isobutyl chloroformate yielding PEG-isoBu anhydride (for synthesis details, see the Experimental section), which was then added dropwise to Ox-(OH)_2_ overnight to obtain Ox-OH-PEG-Es. Ox-SR-PEG-Es was synthesized *via* coupling of SR-717 and Ox-OH-PEG-Es using TBTU comparable to the synthesis of Ox-SR-OAc. This new derivative was adequately soluble for subsequent animal studies. Next, we investigated the hydrolytic stability of Ox-SR-PEG-Es in phosphate buffer (PB) at pH 7.4 and 20 °C over 5 h (Fig. 1). No significant hydrolysis was observed, which is well in line with the stability kinetics observed for other multi-action oxaliplatin(iv)-based prodrugs.^12,37^ The reduction properties were studied by co-incubation with 10 equivalents (eq.) of l-ascorbic acid (AA) as a reducing agent (Fig. 1).

Unexpectedly, the reduction of Ox-SR-PEG-Es was notably fast, with only traces of the original prodrug remaining after ∼3 h of incubation. This reductive instability of Ox-SR-PEG-Es contradicted the slow reduction of dual-action oxaliplatin(iv) prodrugs bearing *e.g.* aspirin, which contains a similar acetyl benzoic acid motif.^37^

We were interested in elucidating the unexpected reduction behavior of Ox-SR-PEG-Es in more detail and investigated the reduction kinetics of a panel of four reference platinum(iv)-acetato complexes containing simplified benzoic acid (Bz) derivatives with alternating substitution patterns in accordance with SR-717: Ox-Bz (Bz = benzoic acid), Ox-Bz-F_2_ (Bz-F_2_ = 3,4-difluorobenzoic acid), Ox-Bz-F_2_-NH_2_ (Bz-F_2_-NH_2_ = 2-amino-4,5-difluorobenzoic acid) and Ox-Bz-F_2_-NHAc (Bz-F_2_-NHAc = 2-acetamido-4,5-difluorobenzoic acid, Fig. S1†). Ox-Bz and Ox-Bz-F_2_ were essentially stable in the presence of AA (approximately 95% left after 5 h), which ruled out that the fluorine substituents are responsible for the fast reduction of Ox-SR-PEG-Es. Interestingly, while *ortho*-amination in Ox-Bz-F_2_-NH_2_ marginally increased the rate of reduction compared to Ox-Bz-F_2_ (approximately 80% left), acetylation of the *ortho*-amine (as in Ox-Bz-F_2_-NHAc) severely impacted the reductive stability, with only 40% of the original compound remaining after 5 h. Although aromatic amines are electron-donating substituents, acetamide-based substituents are electron-withdrawing, which can explain the faster reduction of Ox-Bz-F_2_-NHAc over Ox-Bz-F_2_-NH_2_, although the extent of the effect is unexpected. This trend is in line with the differences in the calculated p*K*_a_ values between Bz-F_2_-NHAc (3.1) and Bz-F_2_-NH_2_ (4.5).^38^ Also the Hammet substituent constants *σ*_m_ and *σ*_p_ highlight the stronger electron-withdrawing properties of –NHAc groups (*σ*_m_: 0.00; *σ*_p_: 0.21) over –NH_2_ groups (*σ*_m_: −0.66; *σ*_p_: −0.16), although these parameters cannot be directly used to explain the influence of the *ortho* substituent, because of *e.g.* steric effects.^39^ These structure–activity relationships correlate well with the findings of Choi and coworkers that the reduction of platinum(iv) complexes by ascorbate is strongly influenced by the electron-withdrawing power of the axial ligand.^40^ Thus, the data of the reference complexes suggest that the 3-(1*H*-imidazol-1-yl)pyridazine subunit of SR-717 is responsible for the fast reduction of Ox-SR-PEG-Es.

**Fig. 2:**
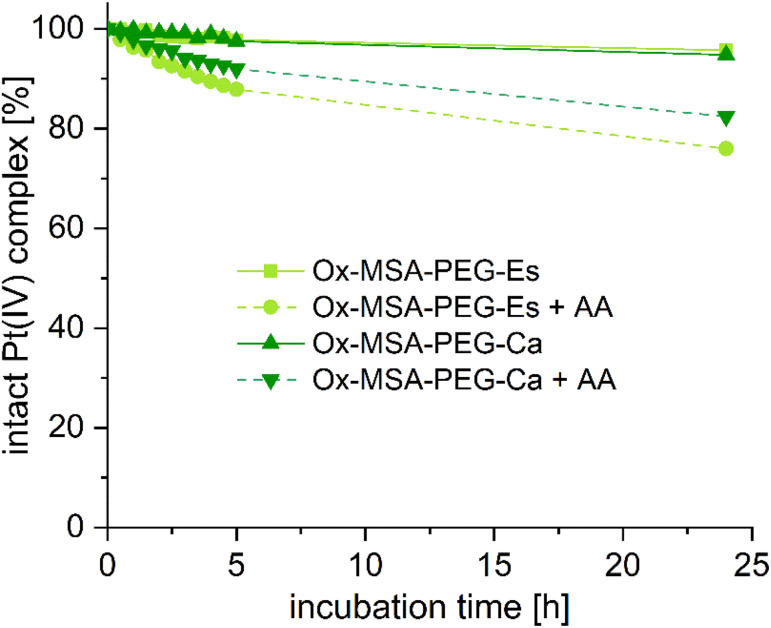
Stability and reduction kinetics of 1 mM Ox-MSA-PEG-Es and Ox-MSA-PEG-Ca in 150 mM PB (pH = 7.4) at 20 °C with or without 10 eq. of AA over 24 h, measured with UHPLC.

Consequently, we focused on the synthesis of platinum(iv) complexes containing the STING agonist MSA-2. MSA-2 was prepared according to the literature,^29^ and, based on the inadequate aqueous solubility of Ox-SR-OAc, we decided to directly synthesize Ox-MSA-PEG-Es*via* a similar synthetic pathway as described for Ox-SR-PEG-Es (Scheme 1). Ox-MSA-PEG-Es was stable in PB at pH 7.4 and 20 °C. However, in contrast to the fast-reducing Ox-SR-PEG-Es, ∼80% of Ox-MSA-PEG-Es remained intact after 24 h of incubation in the presence of 10 eq. of AA (Fig. 2).

In comparison, we also synthesized the respective carbamate analogue Ox-MSA-PEG-Ca. Therefore, Ox-(OH)_2_ was activated asymmetrically *via* dropwise addition of the peptide-coupling reagent *N*,*N*-disuccinimidyl carbonate (DSC) overnight, and subsequently 3,6,9,12-tetraoxatridecanamine (PEG-NH_2_) was added to obtain Ox-OH-PEG-Ca. Finally, MSA-2 was coupled with TBTU to yield Ox-MSA-PEG-Ca. The stability and reduction kinetics of Ox-MSA-PEG-Ca were very well comparable to Ox-MSA-PEG-Es (Fig. 2), which is in agreement with the structure–activity relationships described earlier.^41^

### *In vitro* anticancer activity of platinum(iv) complexes

**Fig. 3:**
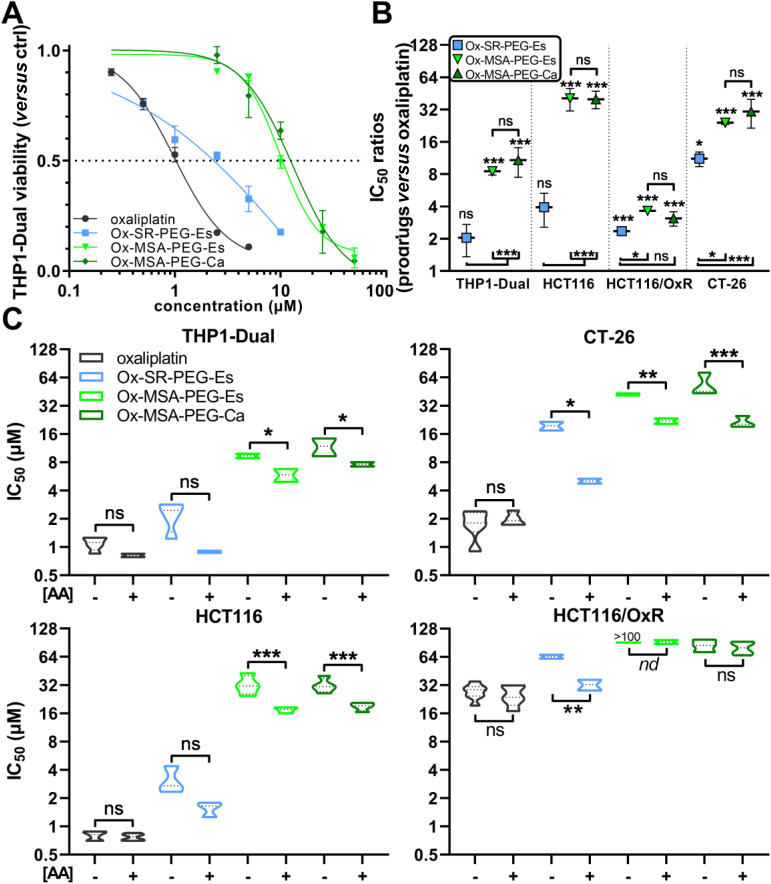
Cytotoxic activity of platinum(iv) prodrugs and oxaliplatin in various cancer cell models and the impact of AA. (A–C) Impact of 72 h of drug exposure on the viability of cancer cell lines, determined by an MTT-based assay. (A) Dose–response curves of the indicated platinum(iv) prodrugs and oxaliplatin for the human THP1-Dual monocytic cell model. Dose–response curves were modelled using a four parameter logistic (4PL) non-linear regression model. One representative experiment out of at least three performed is shown. (B) IC_50_ ratios, derived from the IC_50_ values listed in Table S1,† of the platinum(iv) prodrugs and oxaliplatin in the indicated cell models. Data points in (A) and (B) are shown as mean ± SD. (C) Impact of co-incubation with AA (50 μM) on the IC_50_ values (μM) of the indicated drugs. For underlying values compare Table S1.† Data in (B) and (C) are derived from at least three independent experiments in triplicate. The statistical significance of differences in IC_50_ ratios between prodrugs and oxaliplatin (B), and between –AA and + AA (C) was tested using one-way ANOVA (with the Tukey (B) or Sidak (C) multiple comparison test). IC_50_ = half-maximal inhibitory concentration; AA = l-ascorbic acid; ns = non-significant; and nd = not determinable. *p < 0.05; **p < 0.01; and ***p < 0.001.

A key characteristic of platinum(iv) prodrugs is their reduced cytotoxicity *in vitro* caused by the necessity for activation by reduction. Hence, the reducibility under biological conditions is an important parameter for cellular accumulation and efficacy *in vitro*. Our group previously published oxaliplatin(iv) complexes with greatly reduced cytotoxicity compared to oxaliplatin *in vitro*, but superior antitumor efficacy *in vivo*.^5,12,13^ To elucidate the impact of the observed differences in the reduction properties (compare Fig. 1 and 2) on the anticancer activity, the respective platinum(iv) complexes Ox-SR-PEG-Es, Ox-MSA-PEG-Es and Ox-MSA-PEG-Ca were studied in detail on cancer cells *in vitro*. The monocytic THP1-Dual and colorectal cancer cell models of human (HCT116 and HCT116/OxR) and murine (CT-26) origin were used to compare the impact of the platinum(iv) compounds on cell viability in comparison with oxaliplatin, SR-717 and MSA-2 after continuous exposure for 72 h. Oxaliplatin exhibited cytotoxic activity in the low μM range in THP1-Dual, HCT116 and CT-26 cells, indicating sensitivity towards platinum(ii)-based chemotherapy. Interestingly, distinct differences in the cytotoxic activities were observed for the investigated platinum(iv) prodrugs (Fig. 3, for all IC_50_ values, see Table S1†).

Ox-SR-PEG-Es exhibited the lowest reduction of cytotoxic activity at the IC_50_ level as compared to free oxaliplatin, ranging from a 2.0-fold (THP1-Dual) to a maximum 11.1-fold (CT-26) difference (Fig. 3B). In contrast, Ox-MSA-PEG-Es and Ox-MSA-PEG-Ca were distinctly less active compared to Ox-SR-PEG-Es, indicated by elevated IC_50_ ratios up to ∼40-fold (Fig. 3B). This observation became evident not only in hematogenic THP1-Dual cells but also in all human and murine colorectal cancer models investigated. Comparable cytotoxic activity between oxaliplatin and Ox-SR-PEG-Es and the massively reduced cytotoxicity of Ox-MSA-PEG-Es and Ox-MSA-PEG-Ca are well in agreement with their stability and reduction kinetics (compare Fig. 1 and 2). Interestingly, the loss of cytotoxic activity of the prodrugs was markedly lower in HCT116/OxR cells as compared to the other models, presumably due to reduced drug accumulation as a consequence of acquired oxaliplatin resistance.^42^ The oxaliplatin-resistant HCT116/OxR cell line exhibited a 34.3-fold decrease in vulnerability to free oxaliplatin as compared to the parental HCT116 cells (IC_50_: 27.5 μM *vs.* 0.8 μM), which is in agreement with previous reports.^13^ Distinctly reduced resistance factors were found for both Ox-MSA-PEG-Es (>3.1-fold) and Ox-MSA-PEG-Ca (2.7-fold), while a markedly higher factor of 20.5 was observed for Ox-SR-PEG-Es (Fig. S2†). This is again in line with the insufficient complex stability of Ox-SR-PEG-Es under cell culture conditions. Furthermore, reduced resistance levels for the platinum-MSA-2 complexes indicate that stable prodrugs are less affected by the respective resistance mechanisms of HCT116/OxR cells. In addition, all tested cell lines were co-incubated with AA to test the impact of reduction of the platinum(iv)-prodrugs on cell viability. Indeed, this resulted in increased cytotoxic activity for the three platinum(iv)-complexes in all tested cell lines compared to the “non-reducing” conditions (Fig. 3C).

Finally, combination experiments were performed to investigate whether the two released pharmacologically active compounds, STING agonists and oxaliplatin, interact regarding cytotoxicity (Fig. S3†). Of note, none of the investigated cell lines exhibited vulnerability to the free single agent SR-717 or MSA-2 (IC_50_ > 75 μM, Fig. S3A and B†), although STING and the downstream effectors TANK-binding kinase 1 (TBK1) and interferon regulatory factor 3 (IRF-3) are expressed in all cases (Fig. S4†). In the combination setting, we could identify a weak synergism between STING activation and oxaliplatin cytotoxicity solely in the monocytic THP1-Dual cell model, while no interactions were found in the case of the colorectal cancer cell lines tested (Fig. S3C†).

### Intracellular accumulation of platinum(iv) complexes

**Fig. 4:**
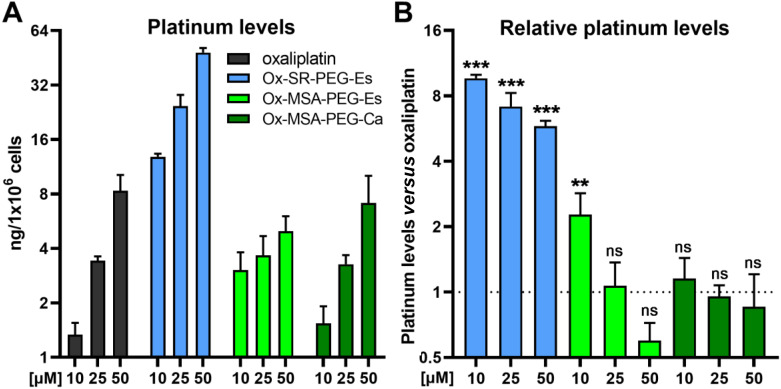
Cellular platinum content in THP1-Dual cells treated with platinum(iv) prodrugs as compared to oxaliplatin, measured with ICP-MS. (A and B) Cells were exposed to the indicated concentrations of the investigated drugs for 3 h and cellular platinum accumulation was measured by ICP-MS and normalized to the cell number. (A) Cellular platinum levels in THP1-Dual cells. (B) Relative prodrug-derived platinum levels normalized to oxaliplatin at equimolar concentrations. Data points in A and B are shown as mean ± SD. The statistical significance of differences in relative platinum levels between prodrugs and oxaliplatin (B) was tested using one-way ANOVA (with the Dunnett multiple comparison test). ns = non-significant. **p < 0.01; ***p < 0.001.

In the next step, the cellular accumulation of the platinum(iv) complexes was investigated in comparison with oxaliplatin. THP1-Dual cells were exposed to varying drug concentrations for 3 h and the intracellular platinum content was measured with ICP-MS (Fig. 4). Oxaliplatin accumulated dose-dependently, with intracellular platinum levels consistent with literature reports (Fig. 4A).^13^ While treatment with Ox-SR-PEG-Es resulted in significantly higher platinum levels compared to oxaliplatin, both Ox-MSA-PEG-Es and Ox-MSA-PEG-Ca did not show a comparable amount of platinum hyper-accumulation. These data strongly suggest that Ox-SR-PEG-Es is taken up as the intact platinum(iv) complex within the incubation time of 3 h, despite its comparably high instability, and not as released oxaliplatin. A higher degree of lipophilicity of Ox-SR-PEG-Es compared to both Pt-MSA complexes is indicated by their HPLC retention times of 4.52 min for Ox-SR-PEG-Es, 3.42 min for Ox-MSA-PEG-Es and 3.36 min for Ox-MSA-PEG-Ca, respectively, and provides a plausible explanation for the observed enhanced accumulation.

In addition to the clear-cut hyper-accumulation of Ox-SR-PEG-Es, low doses of Ox-MSA-PEG-Es (10 μM) resulted in a significantly higher platinum content in comparison with oxaliplatin as well (Fig. 4B). Interestingly, Ox-MSA-PEG-Es treatment resulted in lower intracellular platinum levels at the highest tested dose (50 μM) compared to oxaliplatin, albeit without statistical significance. In contrast, Ox-MSA-PEG-Ca was accumulating comparable to oxaliplatin at all dose levels. These data prove that minor changes in the design of platinum(iv) complexes, *e.g.* changing the axial ester linkage to a carbamate, can impact the accumulation dynamics. However, this 3 h short-term cellular accumulation of the platinum compounds does not reflect their cytotoxic activity in 72 h viability assays (compare Fig. 3).

### STING-dependent IRF and NF-κB activation by platinum(iv) complexes

**Fig. 5:**
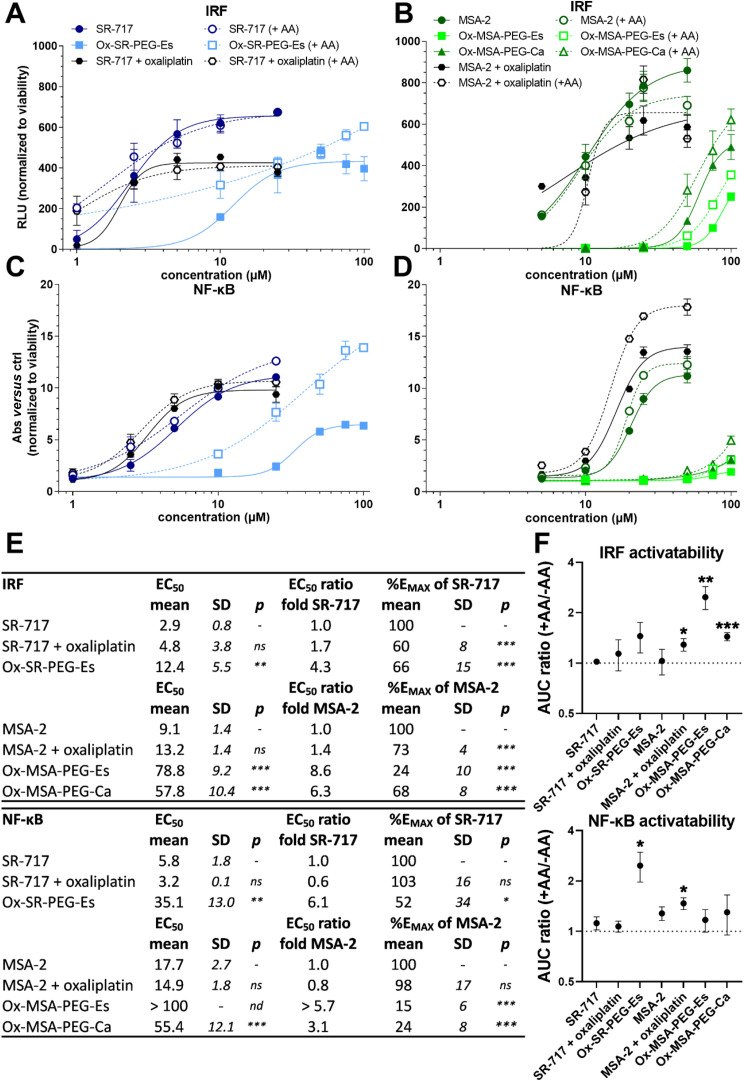
IRF and NF-κB pathway induction by platinum(iv) prodrugs as compared to reference compounds and the impact of reducing conditions. IRF-inducible luciferase (A and B), as well as NF-κB-inducible secreted embryonic alkaline phosphatase (SEAP) reporter levels (C and D) were measured in the THP1-Dual cell culture supernatant following 24 h of exposure to the indicated drugs or the combinations. Oxaliplatin was always applied at 5 μM. (A–D) Dose–response curves, modelled using a four parameter logistic (4PL) non-linear regression model, were normalized to cell viability (Fig. S6†). Impact of reducing conditions were tested by co-incubation with AA (50 μM). One representative experiment out of at least three performed is shown. (E) EC_50_ values and EC_50_ ratios (prodrug/free STING agonist), as well as the maximal induction of the prodrugs in the percent of the maximal response of the single agent SR-717 or MSA-2 (%*E*_MAX_), derived from dose–response curves in (A–D) are depicted for IRF and NF-κB as indicated. (F) Impact of reducing conditions is calculated as the area under curve (AUC) ratio of +AA and −AA. Data points in (A–D) and (F) are shown as mean ± SD. Data are derived from at least three independent experiments in triplicate. The statistical significance of differences in the EC_50_ and *E*_max_ values between the indicated combination groups and the respective free STING agonist (SR-717 or MSA-2) was tested using one-way ANOVA (with the Dunnett multiple comparison test) (*p* values are given). The statistical significance of differences in the AUC between +AA and −AA in (F) was tested using Student's *t*-test. IRF = Interferon regulatory factor; NF-κB = Nuclear factor kappa-light-chain-enhancer of activated B cells; RLU = relative light units; Abs = absorbance; ctrl = control; EC_50_ = half-maximal effective concentration; AA = l-ascorbic acid; AUC = area under the curve; ns = non-significant; and nd = not determinable. *p* < 0.05; **p < 0.01; and ***p < 0.001.

Next, we investigated the impact of the reductive release of STING agonists from platinum(iv) prodrugs on IRF and NF-κB pathway activation in the THP1-Dual reporter cell model in comparison to free oxaliplatin, SR-717 and MSA-2 after 24 h of exposure. This reporter cell model enables simultaneous monitoring of activation of both pathways *via* the detection of secreted reporters. In general, SR-717 and MSA-2 both robustly activated the IRF as well as the NF-κB signaling pathway (Fig. 5A–D). In the case of SR-717, a half-maximal effective concentration (EC_50_) of 2.9 μM (±0.8) was found to trigger the IRF pathway, which is well in agreement with previous reports (Fig. 5E, EC_50_: 2.1 μM).^28^ Furthermore, application of MSA-2 (EC_50_: 9.1 μM) revealed a ∼3.1-fold lower IRF-inducing activity when compared to SR-717-treated cells (Fig. 5E), which can partially be explained by the required dimerization of MSA-2 for STING activation.^29^ Oxaliplatin monotherapy did not induce observable activation of IRF or NF-κB signaling pathways (Fig. S5†). Interestingly, this finding contrasts earlier reports suggesting that platinum-induced DNA damage is capable of triggering the STING and NF-κB pathways.^43^

All three platinum(iv) prodrugs were able to significantly stimulate IRF and NF-κB signalling (Fig. 5A–D). Ox-SR-PEG-Es revealed a ∼4.3-fold attenuated activity (EC_50_: 12.4 μM) for the IRF induction in comparison with free SR-717, with a maximal response (*E*_MAX_) of 66% relative to the maximal induction achieved by SR-717 (Fig. 5E). In the case of the NF-κB pathway, EC_50_ values were more than doubled as compared to the IRF pathway. A distinctly decreased IRF activation was also observed for Ox-MSA-PEG-Es (8.6-fold) and Ox-MSA-PEG-Ca (6.3-fold) as compared to free MSA-2. Notably, while Ox-MSA-PEG-Ca achieved a response of 68% relative to the maximum IRF induction by MSA-2 at the highest tested dose (100 μM), only 24% was achieved by treatment with Ox-MSA-PEG-Es. This trend towards a diminished relative potency was also found in the case of NF-κB activation (%*E*_MAX_Ox-MSA-PEG-Ca*vs.*Ox-MSA-PEG-Es: 24% *vs.* 15%). These observations were surprising, given that the two compounds showed comparable AA-induced reduction rates (Fig. 2) which should result in similar amounts of released STING agonist. Nevertheless, despite these differences in downstream signal pathway activation, both MSA-2-containing complexes exerted comparable cytotoxicity after 72 h (Fig. 3).

To establish the STING-dependent specificity of the observed IRF/NF-κB activation, similar studies were conducted with co-administration of the STING inhibitor H-151 (Fig. S6†). Notably, the IRF and NF-κB reporter signals were completely abolished in the presence of the inhibitor, clearly demonstrating that STING-dependent pathway activation occurs with both free STING agonists and STING agonist-releasing platinum(iv) prodrugs.

AA supplementation significantly enhanced the IRF-induced reporter signals for Ox-MSA-2 complexes, especially Ox-MSA-PEG-Es, compared to the non-reductive setting. In contrast, no notable modulation was observed for Ox-SR-PEG-Es, SR-717, MSA-2, or combinations of SR-717 and oxaliplatin. The combination of oxaliplatin and MSA-2 in the presence of AA moderately but significantly raised reporter levels, which was not found with the administration of the individual drugs (Fig. 5F). Of note, the particularly higher STING activation tendency of Ox-MSA-PEG-Es in the presence of AA can be explained by the inherently low downstream pathway-inducing activity (%*E*_MAX_ of MSA-2: 24%) compared to Ox-MSA-PEG-Ca (%*E*_MAX_ of MSA-2: 68%). Reducing conditions significantly raised NF-κB-induced reporter levels for Ox-SR-PEG-Es and, to a lesser extent, for the combination of MSA-2 and oxaliplatin (Fig. 5F).

Prodrug activation results in the concomitant liberation of SR-717/MSA-2 and oxaliplatin, which could potentially trigger or enhance pathway activation itself.^43^ Hence, reporter activations were investigated following administration of free oxaliplatin in combination with SR-717 or MSA-2. While the IRF reporter activity of oxaliplatin combinations reached a maximal response rate (%*E*_MAX_) of 60% and 73% relative to the free STING agonists SR-717 and MSA-2, respectively, induction of the NF-κB pathway revealed %*E*_MAX_-values of 103% and 98% (Fig. 5). Reporter induction was in all cases normalized to cell viability as determined by MTT assays, considering that platinum-induced cytotoxicity can attenuate reporter levels by reducing viable cell numbers (Fig. S6E–H†). Given that normalized *E*_MAX_ values remained distinctly below 100% in the case of IRF induction, an inhibitory effect of oxaliplatin especially for STING-mediated IRF pathway activation can be postulated.

Taken together, MSA-2-containing prodrugs presented favorable reduction and activation kinetics and hence were chosen for further in-depth analyses.

### Induction of DNA damage and cytokine/chemokine secretion by platinum(iv) complexes

In parallel to IRF- and NF-κB-induced reporter activation, regulation of STING-downstream pathways in the THP1-Dual reporter cell line was studied at the protein level with immunoblots and the results were compared to DNA damage and cell death markers (p-H2A.X, cleaved caspase-3, and cleaved PARP) typically induced by oxaliplatin (Fig. S7†). STING is phosphorylated by TBK1 at Ser366, which leads to the activation of NF-κB (p65) and, subsequently, upregulation of type I IFN and IFN-regulated immune response genes such as ISG15.^44^ Phosphorylation of STING indicated on-target pathway activation.^45^ Elevated phosphorylation of NF-κB was only detected in cells treated with MSA-2 and not with the platinum-MSA-2 prodrugs. Induction of ISG15 was only triggered in cells treated with MSA-2 and Ox-MSA-PEG-Ca, with the latter especially upon AA supplementation. Taken together, this confirmed the activation of the STING signaling axis by the investigated compounds. Free MSA-2, Ox-MSA-PEG-Es and Ox-MSA-PEG-Ca induced DNA damage, indicated by upregulation of the DNA damage marker p-H2A.X, as well as cleavage of caspase-3 and PARP. Additionally, DNA damage-inducing activity was distinctly increased for the prodrugs in AA-treated cells, demonstrating enhanced release of the bioactive ligands under reductive conditions (Fig. S7†).

We additionally monitored target gene activation downstream of STING at the mRNA level. MSA-2 distinctly induced the immune response genes interferon-beta (IFN-β), ISG15, IFIT1, CXCL10 and CCL5, confirming downstream STING target gene activation (Fig. S8†). Interestingly, Ox-MSA-PEG-Es exerted a low propensity towards the activation of the investigated genes, while Ox-MSA-PEG-Ca distinctly raised transcript levels to a comparable extent as free MSA-2 (Fig. S8A†). The overall higher activation capabilities under reductive conditions (±AA) of Ox-MSA-PEG-Es in comparison with Ox-MSA-PEG-Ca (Fig. S8B†) corroborate our findings concerning IRF pathway induction (compare Fig. 5).

Furthermore, secretion of type I interferons was monitored in cell culture supernatants. Treatment of THP1-Dual cells with MSA-2 and Ox-MSA-PEG-Ca induced distinct secretion of cytokines and chemokines, which is consistent with STING pathway activation at the RNA and protein levels (Fig. S9†). Most pronounced upregulations were found in the case of the chemokines CCL5 and CXCL10, which were both secreted at similar levels upon treatment with MSA-2 or Ox-MSA-PEG-Ca. In addition, MSA-2 as well as MSA-2-bearing prodrugs induced elevation of IFN-α and to some extent IL-1a, while TNF-α and especially IFN-γ were activated predominantly by oxaliplatin.

### Synthesis and albumin-binding kinetics of platinum(iv)–maleimide complexes

**Scheme 2:**
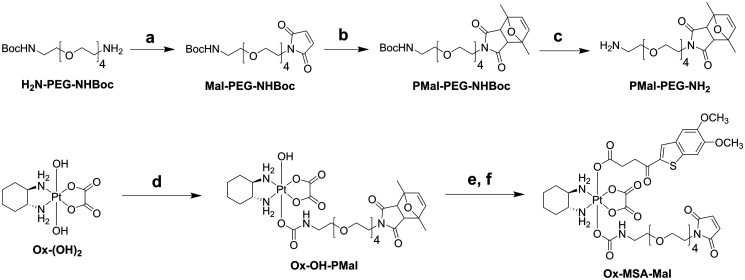
Synthetic route to Ox-MSA-Mal; (a) methyl 2,5-dioxo-2,5-dihydro-1*H*-pyrrole-1-carboxylate and saturated NaHCO_3_ solution at 0 °C and RT; (b) 2,5-dimethylfurane and ACN at 60 °C; (c) 1.25 M HCl and EtOH at RT; (d) PMal-PEG-NH_2_, DSC, and DMF at RT; (e) DMF at 90 °C; (f) MSA-2, TBTU, TEA, and DMF at RT.

Albumin binding can strongly improve the anticancer activity of dual-action platinum(iv) prodrugs.^13,37^ The thorough *in vitro* characterization study revealed MSA-2-bearing platinum(iv) complexes as promising candidates for maleimide-functionalization and subsequent *in vivo* testing in mouse models. Given that the carbamate compound Ox-MSA-PEG-Ca presented overall favorable properties in terms of stability and downstream signal activation as compared to the ester analogue Ox-MSA-PEG-Es (compare Fig. 2, 5 and S7, S8†), the albumin-binding complex Ox-MSA-Mal was synthesized utilizing a carbamate linkage between the maleimide ligand and the platinum(iv) core (Scheme 2). In our previous projects, the PEG-maleimide was introduced as a second axial ligand *via* an isocyanate intermediate prepared from the commercially available carboxylic acid.^37^ Unfortunately, the yield was low and also attempts to synthesize Ox-OH-Mal were not promising. We recently published a new synthesis strategy towards carbamate-linked, PEG-maleimide-containing oxaliplatin(iv) complexes using dimethyl furane-protected maleimides and employing the coupling agent DSC in a similar fashion to that described for Ox-OH-PEG-Ca (Scheme 1).^46^ With this strategy the precursor complex Ox-OH-PMal could be successfully synthesized. Consecutive maleimide deprotection *via* a retro-Diels–Alder reaction at 90 °C and attachment of MSA-2 using TBTU afforded the final complex Ox-MSA-Mal.

**Fig. 6:**
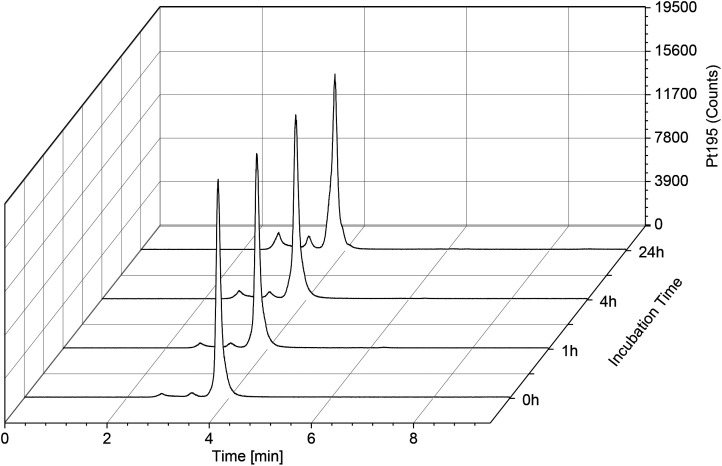
^195^Pt-traces of incubation of 50 μM Ox-MSA-Mal in FCS (containing 150 mM PB, pH = 7.4) at 37 °C over 24 h, measured by SEC-ICP-MS.

Subsequently, we focused on investigating the albumin binding properties of Ox-MSA-Mal compared to Ox-MSA-PEG-Ca by incubation in phosphate-buffered fetal calf serum (FCS) at 37 °C over 24 h. ^195^Pt traces were measured *via* size-exclusion chromatography-inductively coupled plasma mass spectrometry (SEC-ICP-MS), for which the results are presented in Fig. 6. Serum proteins have a retention span of ∼2–4 min as part of the high-molecular weight fraction with albumin at ∼4 min (Fig. S10†). After incubation with Ox-MSA-Mal, approximately 99% of the platinum was already bound to albumin at the 0 h time point and the drug conjugate was stable over 24 h without any release of platinum from the protein. In contrast, the reference complex Ox-MSA-PEG-Ca lacked the ability to conjugate to albumin (Fig. S11†) and was majorly found in the low-molecular weight fraction, even after an extended incubation time up to 24 h, with only traces of the compound detected in the protein fraction.

### Serum pharmacokinetics and organ distribution of platinum(iv) complexes *in vivo*

**Fig. 7:**
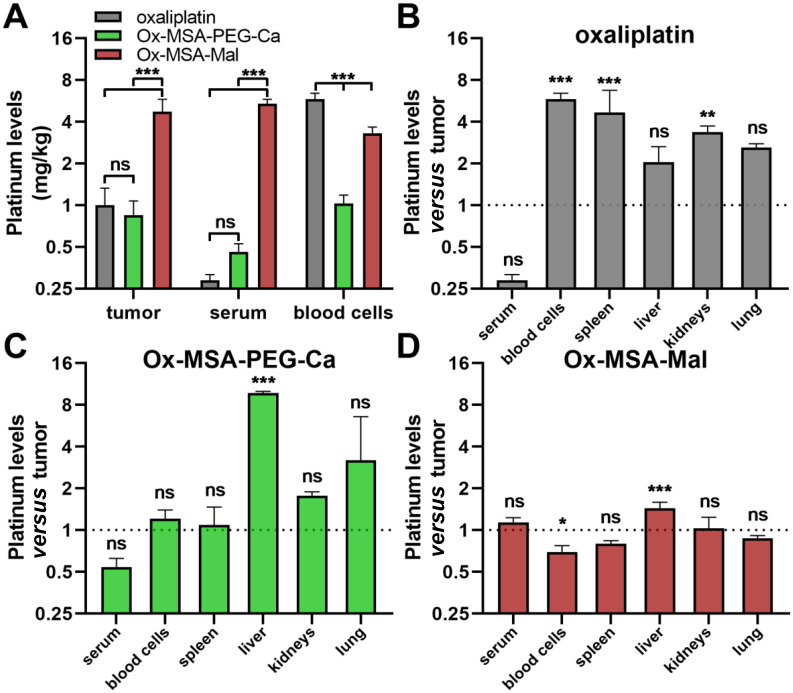
Tumor and organ platinum contents following treatment with MSA-2-releasing platinum(iv) prodrugs as compared to free oxaliplatin. (A–D) CT-26 colorectal cancer-bearing BALB/c mice (*n* = 4 per group) were dosed twice (day 1 and 4) with the indicated drugs in all cases equimolar to 9 mg kg^−1^ oxaliplatin. Animals were euthanized 24 h after the second dose and platinum levels were determined by ICP-MS. Data points are shown as mean ± SD. (A) Absolute platinum contents in tumor, serum and blood cells are depicted. (B–D) For each indicated compound, platinum levels in the respective organs are given normalized to the corresponding tumor tissue. The statistical significance between the applied drugs (A) or between tumor and the respective organs (B–D) was tested by one-way-ANOVA (with the Tukey multiple comparison test). ns = non-significant. *p < 0.05; **p < 0.01; and ***p < 0.001.

Of note, cell culture assessment of albumin-targeted prodrugs is not feasible due to interactions of the maleimide moiety with multiple cell culture medium components. Consequently, in the next step, we directly investigated how the endogenous albumin binding of Ox-MSA-Mal impacts its pharmacological behavior and organ distribution in CT-26 tumor-bearing mice in comparison with Ox-MSA-PEG-Ca and oxaliplatin. Mice were treated twice a week by intravenous (i.v.) application and platinum levels were determined in serum, organs and tumor tissues with ICP-MS 24 h after the second dose (Fig. 7). Equimolar doses corresponding to the maximum tolerated dose (MTD) of oxaliplatin (= 9 mg kg^−1^) were administered. Treatment with Ox-MSA-Mal resulted in distinctly elevated platinum levels in the serum and tumor and clearly lowered accumulation in peripheral blood cells as compared to Ox-MSA-PEG-Ca or oxaliplatin-treated animals (Fig. 7A). These findings corroborate previous reports on maleimide-functionalized oxaliplatin(iv) complexes.^5,12–14^ Low serum platinum levels of oxaliplatin- or Ox-MSA-PEG-Ca-treated animals suggest fast systemic excretion and peripheral distribution. Accordingly, platinum levels in organs of oxaliplatin-treated mice were distinctly higher in comparison to tumor platinum contents, reaching significance in the case of the spleen and kidneys (Fig. 7B). Ox-MSA-PEG-Ca was primarily localized to the liver (9.7-fold higher than in the tumor), while strong accumulation in blood cells, spleen, and kidneys was absent (Fig. 7C). In sharp contrast, Ox-MSA-Mal demonstrated only minor liver accumulation (1.4-fold higher than in the tumor) and even lower platinum levels in blood cells (Fig. 7D). This confirms an improved pharmacological profile and enhanced tumor-targeting properties of the albumin-binding prodrug.

### Immune activation induced by platinum(iv) complexes *in vivo*

**Fig. 8:**
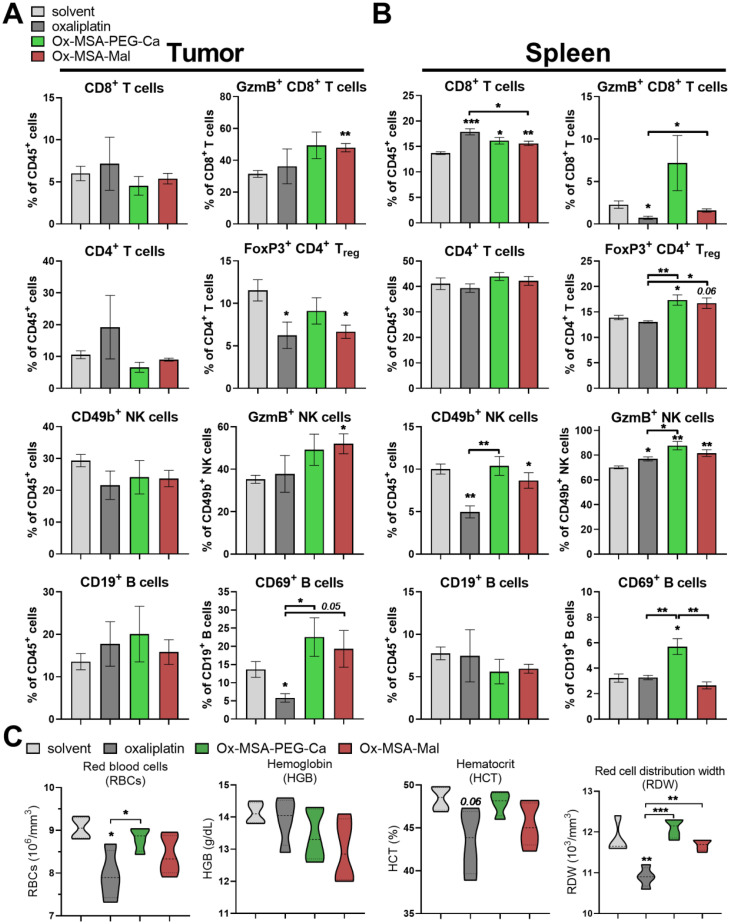
Immune cell activation, tumor infiltration and red blood cell parameters following treatment with Ox-MSA-PEG-Ca and Ox-MSA-Mal as compared to free oxaliplatin. (A–C) CT-26-bearing BALB/c mice (*n* = 4 per group) were dosed twice (day 1 and 4) equimolar to 9 mg kg^−1^ oxaliplatin. Animals were euthanized 24 h after the second dose. Immediately before euthanization, blood was drawn from the submandibular vein and blood reports were established using a Vet ABC hematology analyzer (C). Immune cell populations were quantified in tumor and spleen tissues by flow cytometry (A and B). Frequency of the indicated immune cell populations (as percentage of CD45^+^ cells) and their activation state (as a percentage of the respective immune cell compartment) in (A) tumor and (B) spleen are depicted as mean ± SEM. Representative gates of GzmB^+^ or FoxP3^+^ cells in (A and B) are depicted in Fig. S12A and B.† The gating strategy used to identify the indicated immune cell populations is depicted in Fig. S14.† The statistical significance between the groups was tested by Student's *t*-test. (C) Selected red blood cell parameters from full blood reports are depicted as violin plots (the median and quartiles are shown using bold and dotted lines, respectively). The statistical significance between the groups was tested by one-way-ANOVA (with the Tukey multiple comparison test). *p < 0.05; **p < 0.01; and ***p < 0.001.

Next, we analyzed how treatment with Ox-MSA-PEG-Ca and Ox-MSA-Mal impacts the frequencies of selected immune cell populations and their activation status in spleen and tumor tissue as compared to oxaliplatin 24 h after the second dose (Fig. 8 and S12†). In general, none of the investigated drugs significantly affected the frequency of tumor-infiltrating CD45^+^ immune cells. Oxaliplatin and to a lesser extent Ox-MSA-Mal significantly upregulated CD45^+^ cells among viable splenic cells (Fig. S12C and D†). Neither treatment with oxaliplatin nor MSA-2-containing prodrugs induced changes in the amount of tumor-infiltrating CD8^+^ T cells as compared to solvent-treated animals. In contrast, treatment with all investigated drugs significantly elevated splenic CD8^+^ T cells in the CD45^+^ cell population, with the strongest effect observed for oxaliplatin. Several immune cell activation markers were enhanced predominantly by the platinum(iv) complexes, including granzyme B (GzmB) on CD8^+^ T cells in the tumor and on NK cells both in the spleen and tumor, as well as CD69 on splenic and tumor-infiltrating B cells. Additionally, while treatment with Ox-MSA-PEG-Ca and Ox-MSA-Mal selectively elevated the FoxP3^+^ CD4^+^ T cell compartment in the spleen, indicative of regulatory T cells (T_regs_), reduced levels were observed in the tumor tissue by all investigated compounds (Fig. 8 and S12A, B†). Altogether, these observations suggest that Ox-MSA-PEG-Ca and Ox-MSA-Mal distinctly activate an adaptive immune response. Accordingly, the upregulation of several cytokines and chemokines from the innate immune system was detected in the plasma of treated animals (Fig. S13†). The highest activations were found for IFN-β, IL-6, and M-CSF with Ox-MSA-PEG-Ca, while chemokines CXCL9 and CXCL11 were most strongly activated by Ox-MSA-Mal. These data indicate activation of an anticancer immune cycle by the investigated platinum(iv) compounds with a clear-cut impact of albumin-targeting in the case of Ox-MSA-Mal.

### Hematological analysis of platinum(iv) complexes *in vivo*

Concerning adverse effects, hematological analysis, performed in parallel to the immune phenotyping experiment in CT-26-bearing animals, revealed anemia in the case of oxaliplatin-treated animals but not in Ox-MSA-PEG-Ca- or Ox-MSA-Mal-treated animals (Fig. 8C). Only treatment with oxaliplatin clearly lowered red blood cell counts (RBCs), levels of hematocrit (HCT) and the red cell distribution width (RDW). In contrast, no significant changes were observed concerning white blood cell parameters by any of the investigated compounds (data not shown). This might be based on the relatively short exposure (24 h after the second dosing) to drug treatment in the immune phenotyping experiment. Consequently, the hematotoxic effects of oxaliplatin and Ox-MSA-Mal were investigated in C57BL/6 mice after long-term treatment (72 h after the fourth dosing; Fig. S15†). In this setting, absolute counts of white blood cells (WBCs), lymphocytes (LYM), monocytes (MO) and granulocytes (GRA) were massively lowered by oxaliplatin therapy. In contrast, treatment with Ox-MSA-Mal induced an increase of white blood cells (WBC) and granulocytes (GRA). Thrombocytopenia, indicated by distinctly reduced platelet counts (PLT) following oxaliplatin therapy, was not evident in animals receiving Ox-MSA-Mal. Distinct RBC toxicity of oxaliplatin was confirmed but was strongly reduced in the case of Ox-MSA-Mal (Fig. S15†).

### Anticancer efficacy of platinum(iv) complexes *in vivo*

**Fig. 9:**
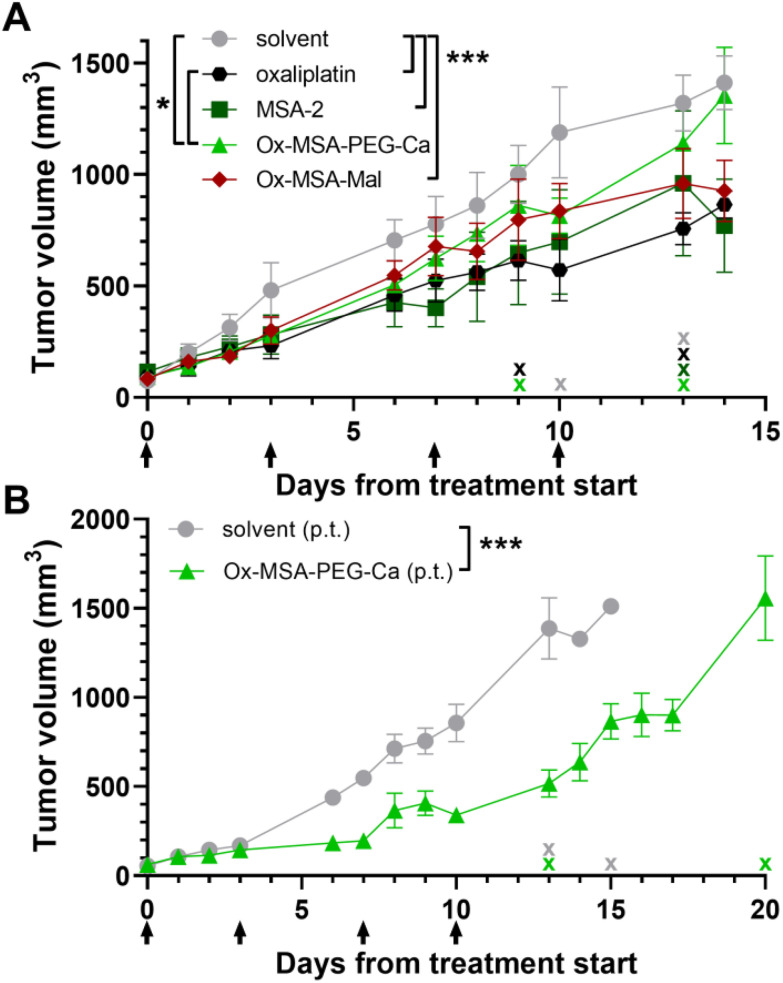
Anticancer efficacy of Ox-MSA-PEG-Ca and Ox-MSA-Mal as compared to free oxaliplatin and MSA-2 and the impact of the application route. (A) CT-26-bearing BALB/c mice (*n* = 4 per group) were dosed twice weekly for two weeks with the indicated drugs equimolar to 9 mg kg^−1^ oxaliplatin. Data points are shown as mean ± SEM. Animals were euthanized upon reaching a tumor volume of >1500 mm^3^, loss in body weight (>20%), tumor ulceration or other indications of deteriorated health following FELASA guidelines. Euthanasia of animals is indicated by an X symbol along the *x*-axis. (A) Tumor volume of animals treated i.v. with the indicated compounds. (B) Tumor volumes of animals treated peritumoral with Ox-MSA-PEG-Ca. The statistical significance of differences in tumor volumes between the treatment groups was tested using two-way ANOVA (with the Tukey multiple comparison test). p.t. = peritumoral. *p < 0.05; ***p < 0.001.

Based on the promising findings in the pharmacological and immune activation experiments above, we next assessed the anticancer efficacy *in vivo* of Ox-MSA-PEG-Ca and Ox-MSA-Mal compared to oxaliplatin and MSA-2. For this purpose, the CT-26 tumor-bearing animals were dosed i.v. twice a week for two weeks at equimolar doses to oxaliplatin (9 mg kg^−1^, Fig. 9A). Generally, the investigated compounds were well tolerated and no significant impact on the weight of the animals was found (Fig. S16A†). Oxaliplatin monotherapy clearly reduced tumor growth (Fig. 9A). MSA-2 also significantly inhibited malignant growth when applied at a low dose of 6.67 mg kg^−1^ (equimolar to 9 mg kg^−1^ of oxaliplatin) *via* the i.v. route, which is well in agreement with previous reports, however using a distinctly higher dose (50 mg kg^−1^) and different application routes (*e.g.* subcutaneous and intratumoral).^29^ Both platinum(iv)-prodrugs significantly reduced tumor growth, with the maleimide-targeted Ox-MSA-Mal exhibiting distinctly stronger anticancer efficacy. However, tumor growth inhibition by Ox-MSA-Mal did not outperform the activity of either of the free components. Considering the higher platinum accumulation in tumors following Ox-MSA-Mal treatment (compare Fig. 7), disadvantageous tumor delivery is unlikely to explain the lack of synergistic effects of the two prodrug components. The rather antagonistic effect of oxaliplatin on MSA-2-mediated STING downstream pathway activation might be considered as one limiting factor (compare Fig. 5). Alternatively, systemic STING agonist application might not reach sufficient levels in the malignant tissue as suggested by the limited immune cell invasion and activation in the tumor in comparison with the spleen (Fig. 8). Consequently, the anticancer activity of Ox-MSA-PEG-Ca was tested in a final experiment with peritumoral (p.t.) application of identical doses used in the i.v. experiment (Fig. 9B). Ox-MSA-Mal could not be employed in this experiment, given the requirement of i.v. administration of maleimide-functionalized prodrugs to enable endogenous albumin-binding. Interestingly, peritumoral application of Ox-MSA-PEG-Ca induced a robust and significant decrease in the tumor volume (Fig. 9B) without negative effects on body weights (Fig. S16B†). These data suggest that i.v. application might indeed lead to insufficient peak levels of free STING agonists inside the complex tumor microenvironment to activate a full anticancer immune cycle.

## Conclusion

Oxaliplatin is a highly effective chemotherapeutic agent acting mainly *via* DNA-damage and stimulation of the immune system but at the same time causing severe side effects that greatly diminish patients’ quality of life. Platinum(iv) prodrugs emerged as promising alternatives due to their enhanced kinetic inertness and higher degree of functionalization to improve tumor efficacy, *e.g. via* the attachment of bioactive ligands or tumor-targeting moieties. In the past decades, albumin emerged as an efficient drug carrier for the enhanced tumor targeting of metal-based drugs either *via* covalent attachment by using maleimide functional groups^47,48^ or by non-covalent interactions.^49^ Our group recently reported several maleimide-functionalized oxaliplatin(iv) prodrugs with distinctly improved anticancer activity over oxaliplatin.^12,14^ The tumor-specific release of platinum(ii) and potentially synergistic, bioactive axial ligands may also mitigate systemic adverse effects of both released compounds and their combination. Since oxaliplatin is considered the strongest inducer of ICD among the approved platinum anticancer drugs^16,17^ and the cGAS-STING pathway plays a major role in anticancer immune recognition,^50^ we decided to synthesize and extensively evaluate oxaliplatin(iv) complexes with activators of the STING pathway. Two STING agonists, SR-717 and MSA-2, with promising antitumor activity were recently published.^28,29^ Hence, in this study, we explored for the first time the synthesis and evaluated the biological properties of a panel of oxaliplatin(iv) prodrugs containing the STING agonists SR-717 or MSA-2. We hypothesized that these complexes should allow synergistic anticancer immune mechanisms and reduce adverse effects characteristic of clinical combination schemes of immune-activating agents and platinum-containing chemotherapy. Indeed, confirming the prodrug concept, platinum(iv) complexes exhibited reduced cytotoxicity, DNA damage, and immune-stimulation *in vitro* as compared to the free parental compounds, with all these parameters re-activated by the presence of the reducing agent AA. This underscored the necessity of complex activation by reduction especially in the case of the more stable Ox-MSA-PEG-Es and Ox-MSA-PEG-Ca complexes. A maleimide-functionalized Pt-MSA-2 derivative formed stable albumin adducts, translating into promising *in vivo* pharmacokinetic profiles with strongly enhanced plasma levels and tumor platinum accumulation in CT-26 tumor-bearing mice in comparison with oxaliplatin and the reference non-maleimide complex. In addition, both Pt-MSA-2 complexes distinctly induced immune activation, evident by significant upregulation of cytotoxic CD8^+^ T cells, NK cell activation and the release of inflammatory cytokines and chemokines, while lacking oxaliplatin-related hematotoxic effects. Interestingly, the albumin-targeted complex Ox-MSA-Mal significantly inhibited colorectal tumor growth *via* the i.v. route, while the non-targeted Ox-MSA-PEG-Ca failed to do so. The latter, however, exerted distinct anticancer activity after peritumoral application, postulating a clear influence of the application route on the efficacy of Pt-MSA complexes. Unfortunately, the promising results in the pharmacological studies of the Ox-MSA-Mal complex did not translate into improved anticancer activity compared to free oxaliplatin or MSA-2. The underlying reasons seem to be complex. Released intratumoral concentrations of free MSA-2 below the activity threshold, due to the slow reduction of Pt-MSA prodrugs, combined with the necessity for ligand dimerization could explain the lack of synergistic activity *via* the i.v. route. This hypothesis is in agreement with a previous study showing that the anticancer activity of i.v. applied liposomal oxaliplatin formulations in colorectal cancer-bearing mouse models was significantly enhanced by intratumoral administration of the STING agonist ADU-S100.^51^ This suggests that achieving sufficiently high intratumoral concentrations of the STING agonist is critical for optimal synergistic anticancer effects. Additionally, the inhibitory effect of oxaliplatin on the MSA-2-mediated STING downstream pathway activity *in vitro* indicates that the simultaneous release of the STING agonist and oxaliplatin from platinum(iv) complexes might also exert antagonistic effects *in vivo*. Moreover, a recent study demonstrated that FOLFOX-activated cGAS-STING signaling mediates the upregulation of the immune checkpoint inhibitor PD-L1, thereby dampening the corresponding anticancer immune response.^43^ Consequently, the combination of Ox-MSA-Mal with PD-1/PD-L1 inhibitory antibodies is currently under investigation. Whether the complex interaction between oxaliplatin and the cGAS-STING signaling pathway is specific to oxaliplatin or extends to other platinum compounds remains to be tested. The group of Guo very recently reported MSA-2-containing cisplatin(iv) prodrugs with distinct anticancer activity *in vivo*, however, at the same time exerting also massively enhanced *in vitro* cytotoxicity as compared to free cisplatin.^33^ This suggests a novel cytotoxic mechanism inherent to this cisplatin(iv) complex.

In conclusion, the strategy of combining a STING activator and oxaliplatin within an albumin-targeted platinum(iv) complex showed notable tumor-inhibitory activity with distinctly reduced adverse effects. However, the obtained antitumor activity was not superior to that of free oxaliplatin or the respective STING agonist. Our current efforts focus on developing strategies to investigate and mitigate the inhibitory effect of oxaliplatin on STING agonist-mediated immune responses to optimize the achieved anticancer effects.

## Experimental section

### Synthesis

#### Materials and methods

Potassium tetrachloridoplatinate was purchased from Johnson Matthey (Switzerland). Water for synthesis was taken from a reverse osmosis system. Reactions were conducted under atmospheric conditions and at room temperature (RT) unless stated otherwise. For HPLC measurements, Milli-Q water (18.2 MΩ cm, Merck Milli-Q Advantage, Darmstadt, Germany) was used. PEG-COOH, H_2_N-PEG-NHBoc and other chemicals and solvents were purchased from commercial suppliers (Sigma-Aldrich, Merck, Acros, Fluka, and Fisher Scientific). SR-717,^19^ MSA-2,^20^ PMal-PEG-NH_2_,^46^ oxaliplatin,^27^ Ox-(OH)_2_ ^23^ and Ox-OH-OAc ^23^ were synthesized as described in the literature. Electrospray ionization (ESI) mass spectra were recorded using a Bruker Amazon SL ion trap mass spectrometer in the positive and/or negative mode by direct infusion at the Mass Spectrometry Centre of the University of Vienna. One- and two-dimensional ^1^H and DEPT ^13^C NMR spectra were recorded using a Bruker AV Neo 500 or AV III 600 spectrometer at 298K. For ^1^H and ^13^C NMR spectra, the solvent residual peak was taken as an internal reference. The ^1^H, DEPT ^13^C NMR spectra and UHPLC chromatograms of the final compounds are depicted in Fig. S17–20.† Purification by preparative reverse phase (RP) HPLC was performed on an Agilent 1200 series system using a Waters XBridge C18 column (19 × 250 mm). Elemental analysis measurements were carried out using a PerkinElmer 2400 CHN elemental analyzer at the Microanalytical Laboratory of the University of Vienna and are within ±0.4%, confirming >95% purity. The contents of TFA and water can vary between different batches of the same compound.

#### (OC-6-44)-acetato-[(1*R*,2*R*)-1,2-cyclohexanediamino]-[2-(6-(1*H*-imidazol-1-yl)pyridazine-3-carboxamido)benzoato]-oxalato-[2,5,8,11-tetraoxatetradecanoato]platinum(iv) (Ox-SR-OAc)

SR-717 (32 mg, 0.09 mmol, 1.5 eq.) was suspended in anhydrous DMSO (2 mL) under an argon atmosphere at RT. Subsequently, TEA (19 μL, 0.14 mmol, 2.3 eq.) and TBTU (35 mg, 0.11 mmol, 1.8 eq.) were added and the reaction mixture was stirred for 30 min at RT. Ox-OH-OAc (29 mg, 0.06 mmol) was added and the reaction mixture was stirred for 20 h at 40 °C. DMSO was removed under high vacuum (50 °C), and the crude mixture was purified by preparative RP-HPLC (25% MeCN (+0.1% CF_3_COOH) in H_2_O (+0.1% CF_3_COOH); isocratic) and lyophilised to afford the title compound as a white solid (30 mg, 61%). ^1^H NMR (500 MHz, DMSO-*d*_6_) *δ* 12.99 (s, 1H, N*H*_Ca_), 9.16–9.04 (s, 1H, C*H*_Ar_), 8.81–8.73 (m, 1H, C*H*_Ar_), 8.72–8.64 (m, 1H, N*H*_2_), 8.62–8.57 (d, *J* = 9.1 Hz, 1H, C*H*_Ar_), 8.54–8.49 (d, *J* = 9.2 Hz, 2H, C*H*_Ar_), 8.43–8.27 (m, 3H, N*H*_2_ + C*H*_Ar_), 8.21–8.13 (t, *J* = 10.3 Hz, 1H, C*H*_Ar_), 7.81–7.66 (m, 1H, N*H*_2_), 7.44 (s, 1H, C*H*_Ar_), 2.83–2.72 (m, 1H, C*H*_cHex_), 2.71–2.61 (m, 1H, C*H*_cHex_), 2.19–2.10 (m, 2H, C*H*_cHex_), 2.01 (s, 3H, PtOCOC*H̲*_3_), 1.65–1.41 (m, 4H, C*H*_cHex_), 1.24–1.06 (m, 2H, C*H*_cHex_) ppm. MS (*m*/*z*): calcd C_25_H_25_F_2_N_7_O_9_NaPt (M + H)^+^, 801.14; found, 801.24.

#### (Isobutyl carbonic)2,5,8,11-tetraoxatetradecan-14-oic anhydride (PEG-isoBu anhydride)

2,5,8,11-Tetraoxatetradecan-14-oic acid (368 mg, 1.56 mmol) was dissolved in anhydrous THF (4 mL) under an argon atmosphere at 0 °C. Subsequently, isobutyl chloroformate (224 μL, 1.71 mmol, 1,1 eq.) and 4-methylmorpholine (179 μL, 1.63 mmol, 1.05 eq.) were added and the reaction mixture was stirred for 15 min at 0 °C, and then for 2 h at RT. Demi-H_2_O (4 mL) and EtOAc (15 mL) were added, the aqueous layer was extracted with EtOAc (3 × 15 mL), and the total organic layers were dried with Mg_2_SO_4_, vacuum filtered and concentrated under reduced pressure to afford the title compound as a light brown oil (485 mg; 93%). ^1^H NMR (500 MHz, DMSO-*d*_6_) *δ* 4.05–4.02 (d, *J* = 6.6 Hz, 2H, C*H̲*_2_CH_i-Pr_), 3.67–3.63 (t, *J* = 6.0 Hz, 2H, C*H̲*_2_OCH_3_), 3.52–3.47 (m, 10H, C*H*_PEG_), 3.44–3.40 (m, 2H, C*H*_PEG_), 3.23 (s, 3H, OC*H*_3_), 2.81–2.78 (t, *J* = 5.9 Hz, 2H, CH_2_C*H̲*_2_COO), 1.98–1.92 (sp, *J* = 6.6 Hz, 1H, C*H*_i-Pr_), 0.92–0.89 (d, *J* = 6.7 Hz, 6H, C*H*_3,i-Pr_) ppm. MS (*m*/*z*): calcd C_15_H_29_O_8_ (M + H)^+^, 337.39; found, 337.42.

#### (OC-6-44)-[(1*R*,2*R*)-1,2-cyclohexanediamino]-hydroxido-oxalato-[2,5,8,11-tetraoxatetradecanoato]platinum(iv) (Ox-OH-PEG-Es)

A solution of PEG-isoBu anhydride (300 mg, 0.89 mmol, 1,1 eq.) in anhydrous DMSO (1 mL) was added overnight to a stirring suspension of Ox-(OH)_2_ (348 mg, 0.81 mmol) in anhydrous DMSO (5 mL) under an argon atmosphere at RT with the aid of a syringe pump. After the final addition, the reaction mixture was stirred for an additional 1 h. DMSO was removed under high vacuum (50 °C), and the crude mixture was purified by preparative RP-HPLC (12% MeCN (+0.1% HCOOH) in H_2_O (+0.1% HCOOH); isocratic) and lyophilized to afford the title compound as a white solid (287 mg, 54%). ^1^H NMR (500 MHz, DMSO-*d*_6_) *δ* 8.61–8.36 (m, 1H, N*H*_2_), 8.21–8.02 (m, 1H, N*H*_2_), 7.91–7.71 (m, 1H, N*H*_2_), 7.20–6.98 (m, 1H, N*H*_2_), 3.61–3.41 (m, 14H, C*H*_PEG_), 3.24 (s, 3H, OC*H*_3_), 2.65–2.45 (m, 2H, C*H*_cHex_, coinciding with the DMSO peak as detected by ^1^H,^1^H-COSY NMR), 2.43–2.38 (t, *J* = 6.6 Hz, 2H, C*H̲*_2_COOPt), 2.12–2.00 (m, 2H, C*H*_cHex_), 1.55–1.40 (m, 3H, C*H*_cHex_), 1.37–1.26 (m, 1H, C*H*_cHex_), 1.18–1.01 (m, 2H, C*H*_cHex_) ppm. MS (*m*/*z*): calcd C_18_H_34_N_2_O_11_NaPt (M + Na)^+^, 672.17; found, 672.22.

#### (OC-6-44)-[(1*R*,2*R*)-1,2-cyclohexanediamino]-[2-(6-(1*H*-imidazol-1-yl)pyridazine-3-carboxamido)benzoato]-oxalato-[2,5,8,11-tetraoxatetradecanoato]platinum(iv) (Ox-SR-PEG-Es)

SR-717 (46 mg, 0.13 mmol, 1.6 eq.) was suspended in anhydrous DMSO (2 mL) under an argon atmosphere at RT. Subsequently, TEA (27 μL, 0.19 mmol, 2 eq.) and TBTU (45 mg, 0.14 mmol, 1.8 eq.) were added and the reaction mixture was stirred for 30 min at RT. Ox-OH-PEG-Es (50 mg, 0.08 mmol) was added and the reaction mixture was stirred for 20 h at 40 °C. DMSO was removed under high vacuum (50 °C), and the crude mixture was purified by preparative RP-HPLC (28% MeCN (+0.1% CF_3_COOH) in H_2_O (+0.1% CF_3_COOH); isocratic) and lyophilised to afford the title compound as a white solid (38 mg, 51%). ^1^H NMR (500 MHz, DMSO-*d*_6_) *δ* 13.02 (s, 1H, N*H*_Ca_), 9.22–9.12 (s, 1H, C*H*_Ar_), 8.80–8.71 (m, 1H, C*H*_Ar_), 8.68–8.62 (m, 1H, N*H*_2_), 8.62–8.57 (d, *J* = 9.1 Hz, 1H, C*H*_Ar_), 8.54–8.49 (d, *J* = 9.2 Hz, 1H, C*H*_Ar_), 8.43–8.27 (m, 3H, N*H*_2_ + C*H*_Ar_), 8.20–8.13 (t, *J* = 10.1 Hz, 1H, C*H*_Ar_), 7.76–7.65 (m, 1H, N*H*_2_), 7.47 (s, 1H, C*H*_Ar_), 3.59–3.55 (t, 2H, PtOCOC*H̲*_2_), 3.52–3.44 (m, 10H, CH_PEG_), 3.44–3.41 (m, 2H, C*H̲*_2_OCH_3_), 3.24 (s, 3H, OC*H*_3_), 2.84–2.74 (m, 1H, C*H*_cHex_), 2.67–2.59 (m, 1H, C*H*_cHex_), 2.57–2.53 (t, 2H, PtOCOCH_2_C*H̲*_2_), 2.21–2.10 (m, 2H, C*H*_cHex_), 1.65–1.55 (m, 1H, C*H*_cHex_), 1.54–2.39 (m, 3H, C*H*_cHex_), 1.20–1.08 (m, 2H, C*H*_cHex_) ppm. ^13^C NMR (126 MHz, DMSO-*d*_6_) *δ* 178.4 (PtO*C*O), 171.2 (PtO*C*O), 163.2 (*C*O_Ox_), 163.2 (*C*O_Ox_), 160.7 (*C*ONH_Ca_), 153.2 (*C*_q,Ar_), 151.8 (*C*_q,Ar_), 150.6 (*C*_q,Ar_), 145.4 (*C*_q,Ar_), 143.8 (*C*_q,Ar_), 136.1 (*C*_q,Ar_), 135.9 (*C*H_Ar_), 129.7 (2x*C*H_Ar_), 120.8 (*C*H_Ar_), 120.1 (*C*H_Ar_), 117.5 (*C*H_Ar_), 108.9 (*C*H_Ar_), 71.2 (*C*H_PEG_), 69.8 (2x*C*H_PEG_), 69.7 (*C*H_PEG_), 69.6 (2x*C*H_PEG_), 69.5 (*C*H_PEG_), 66.8 (*C*H_PEG_), 61.5 (*C*H_cHex_), 60.7 (*C*H_cHex_), 58.0 (O*C*H_3_), 36.4 (PtOCOCH_2_*C̲*H_2_), 31.0 (*C*H_cHex_), 30.6 (*C*H_cHex_), 23.5 (*C*H_cHex_), 23.5 (*C*H_cHex_) ppm. MS (*m*/*z*): calcd C_33_H_41_F_2_N_7_O_13_NaPt (M + Na)^+^, 999.23; found, 999.29. EA calcd C_33_H_41_F_2_N_7_O_13_Pt ·1.5H_2_O: C, 39.49; H, 4.42; N, 9.95. Found: C, 39.24; H, 4.69; N, 10.

#### (OC-6-44)-[(1*R*,2*R*)-1,2-cyclohexanediamino]-oxalato-[4-(5,6-dimethoxybenzo[*b*]thiophen-2-yl)-4-oxobutylcarboxylato]-[2,5,8,11-tetraoxatetradecanoato]platinum(iv) (Ox-MSA-PEG-Es)

MSA-2 (53 mg, 0.18 mmol, 2 eq.) was dissolved in anhydrous DMF (1.5 mL) under an argon atmosphere at RT. TEA (38 μL, 0.27 mmol, 3 eq.) and TBTU (75 mg, 0.23 mmol, 2.5 eq.) were added and the reaction mixture was left stirring for 30 min at RT. Ox-OH-PEG-Es (58 mg, 0.09 mmol) was added and the reaction mixture was left stirring for 24 h in the dark. DMF was removed under high vacuum (40 °C), and the crude mixture was purified by preparative RP-HPLC (30% MeCN (+0.1% CF_3_COOH) in H_2_O (+0.1% CF_3_COOH); isocratic) and lyophilized to afford the title compound as a white solid (54 mg, 65%). ^1^H NMR (500 MHz, DMSO-*d*_6_) *δ* 8.44–8.28 (m, 2H, N*H*_2_), 8.26–8.08 (m, 3H, N*H*_2_+ C*H*_Ar_), 7.60 (s, 1H, C*H*_Ar_), 7.47 (s, 1H, C*H*_Ar_), 3.86 (s, 3H, OC*H*_3_), 3.83 (s, 3H, OC*H*_3_), 3.56–3.51 (t, *J* = 6.5 Hz, 2H, CH_2,MSA_), 3.53–3.40 (m, 12H, C*H*_2,PEG_), 3.33–3.26 (m, 1H, C*H*_PEG_), 3.24–3.16 (m, 4H, OC*H*_3,PEG_ + C*H*_PEG_), 2.73–2.64 (m, 3H + C*H*_cHex_ + C*H*_2_COOPt), 2.65–2.53 (m, 2H, C*H*_2,MSA_, coinciding with the DMSO peak as detected by ^1^H,^1^H-COSY NMR), 2.57–2.51 (m, 1H, C*H*_cHex_), 2.15–2.06 (m, 2H, C*H*_cHex_), 1.57–1.46 (m, 2H, C*H*_cHex_), 1.45–1.30 (m, 2H, C*H*_cHex_), 1.24–1.07 (m, 2H, C*H*_cHex_) ppm. ^13^C NMR (126 MHz, DMSO-*d*_6_) *δ* 192.6 (*C̲*OC_q,Ar_), 178.0 (PtO*C*O), 164.2 (PtO*C*O), 163.4 (*C*O_Ox_), 163.3 (*C*O_Ox_), 150.8 (*C*_q,Ar_), 148.5 (*C*_q,Ar_), 140.6 (*C*_q,Ar_), 135.7 (*C*_q,Ar_), 132.6 (*C*_q,Ar_), 130.3 (*C*H_Ar_), 106.6 (*C*H_Ar_), 104.3 (*C*H_Ar_), 71.2 (*C*H_2,PEG_), 69.8 (2x*C*H_2,PEG_), 69.7 (*C*H_2,PEG_), 69.6 (*C*H_2,PEG_), 69.5 (*C*H_2,PEG_), 66.8 (*C*H_2,MSA_), 60.9 (2x*C*H_cHex_), 58.0 (O*C*H_3,PEG_), 55.9 (O*C*H_3,MSA_), 55.6 (O*C*H_3,MSA_), 36.5 (O*C̲*H_2_CH_2_COOPt), 34.0 (*C*H_2,PEG_), 31.0 (2x*C*H_2,cHex_), 29.7 (*C̲*H_2_COOPt), 23.6 (*C*H_2,cHex_), 23.4 (*C*H_2,cHex_) ppm. MS (*m*/*z*): calcd C_32_H_46_N_2_O_15_NaPt (M + Na)^+^, 948.22; found, 948.29. EA calcd C_32_H_46_N_2_O_15_PtS·0.5TFA: C, 40.33; H, 4.77; N, 2.85; S, 3.26. Found: C, 40.32; H, 4.81; N, 2.78; S, 3.55.

#### (OC-6-34)-[(1*R*,2*R*)-1,2-cyclohexanediamino]-hydroxido-oxalato-[3,6,9,12-tetraoxatridecan-1-amino]platinum(iv) (Ox-OH-PEG-Ca)

A solution of DSC (197 mg, 0.77 mmol, 1.1 eq.) in anhydrous DMSO (3 mL) was added overnight (17 h) to a stirring suspension of Ox-(OH)_2_ (300 mg, 0.7 mmol) in anhydrous DMSO (6 mL) under an argon atmosphere at RT in the dark with the aid of a syringe pump. After the final addition, the reaction mixture was stirred for an additional 1 h. PEG-NH_2_ (216 μL, 1.05 mmol, 1.5 eq.) was added and the reaction mixture was stirred for 24 h at RT in the dark. DMSO was removed under high vacuum (50 °C), and the crude mixture was purified by preparative RP-HPLC (11% MeCN (+0.1% HCOOH) in H_2_O (+0.1% HCOOH); isocratic) and lyophilized to afford the title compound as a white solid (313 mg, 67%). ^1^H NMR (500 MHz, DMSO-*d*_6_) *δ* 9.90–9.46 (bs, 1H, N*H*_2_), 8.40–8.15 (m, 1H, N*H*_2_), 7.77–7.56 (m, 1H, N*H*_2_), 7.21–6.96 (m, 1H, N*H*_2_), 6.35–6.25 + 6.11–5.91 (m, 1H, N*H*_Ca_), 3.55–3.40 (m, 12H, C*H*_PEG_), 3.36–3.30 (m, 2H, C*H*_PEG_), 3.24 (s, 3H, OC*H*_3_), 3.01–2.99 (m, 2H, C*H̲*_2_NH_Ca_), 2.59–2.47 (m, 2H, C*H*_cHex_), 2.17–2.00 (m, 2H, C*H*_cHex_), 1.61–1.40 (m, 3H, C*H*_cHex_), 1.40–1.26 (m, 1H, C*H*_cHex_), 1.19–1.05 (m, 2H, C*H*_cHex_) ppm. MS (*m*/*z*): calcd C_18_H_35_N_3_O_11_NaPt (M + Na)^+^, 687.18; found, 687.22.

#### (OC-6-34)-[(1*R*,2*R*)-1,2-cyclohexanediamino]-oxalato-[4-(5,6-dimethoxybenzo[*b*]thiophen-2-yl)-4-oxobutylcarboxylato]-[3,6,9,12-tetraoxatridecan-1-amino]platinum(iv) (Ox-MSA-PEG-Ca)

MSA-2 (42 mg, 0.14 mmol, 2 eq.) was dissolved in anhydrous DMF (1 mL) under an argon atmosphere at RT. TEA (30 μL, 0.21 mmol, 3 eq.) and TBTU (55 mg, 0.17 mmol, 2.5 eq.) were added and the reaction mixture was left stirring for 30 min at RT. Ox-OH-PEG-Ca (48 mg, 0.07 mmol) was added and the reaction mixture was left stirring for 24 h in the dark. DMF was removed under high vacuum (40 °C), and the crude mixture was purified by preparative RP-HPLC (29% MeCN (+0.1% CF_3_COOH) in H_2_O (+0.1% CF_3_COOH); isocratic) and lyophilized to afford the title compound as a white powder (47 mg, 71%). ^1^H NMR (500 MHz, DMSO-*d*_6_) *δ* 9.62–9.53 + 9.42–9.26 (m, 1H, N*H*_2_), 8.78–8.62 (m, 1H, N*H*_2_), 8.31–8.08 (m, 3H, N*H*_2_+ C*H*_Ar_), 7.60 (s, 1H, C*H*_Ar_), 7.47 (s, 1H, C*H*_Ar_), 6.78–6.71 + 6.32 (m, 1H, N*H*_Ca_), 3.85 (s, 3H, OC*H*_3_), 3.83 (s, 3H, OC*H*_3_), 3.53–3.41 (m, 12H, C*H*_PEG_), 3.55–3.40 (m, 2H, C*H*_PEG_), 3.37–3.15 (m, 7H, C*H*_2,MSA_ + C*H*_PEG_ + OC*H*_3,PEG_), 3.10–2.94 (m, 2H, C*H*_2_NH_Ca_), 2.75–2.53 (m, 4H + C*H*_cHex_ + CH_2,MSA_), 2.18–2.05 (m, 2H, C*H*_cHex_), 1.57–1.45 (m, 2H, C*H*_cHex_), 1.46–1.27 (m, 2H, C*H*_cHex_), 1.26–1.08 (m, 2H, C*H*_cHex_) ppm. ^13^C NMR (126 MHz, DMSO-*d*_6_) *δ* 192.3 (*C̲*OC_q,Ar_), 179.9 (PtO*C*O), 164.2 (*C*ONH_Ca_), 163.4 (*C*O_Ox_), 163.3 (*C*O_Ox_), 150.8 (*C*_q,Ar_), 148.6 (*C*_q,Ar_), 140.6 (*C*_q,Ar_), 135.7 (*C*_q,Ar_), 132.5 (*C*_q,Ar_), 130.3 (*C*H_Ar_), 106.5 (*C*H_Ar_), 104.3 (*C*H_Ar_), 71.3 (2x*C*H_2,PEG_), 69.8 (2x*C*H_2,PEG_), 69.6 (2x*C*H_2,PEG_), 69.2 (2x*C*H_2,PEG_), 60.9 (*C*H_cHex_), 60.7 (*C*H_cHex_), 58.0 (O*C*H_3,PEG_), 55.8 (O*C*H_3,MSA_), 55.6 (O*C*H_3,MSA_), 40.7 (*C̲*H_2_NH_Ca_), 34.0 (*C*H_2,MSA_), 31.0 (*C*H_2,cHex_), 30.9 (*C*H_2,cHex_), 29.6 (*C*H_2,MSA_), 23.7 (*C*H_2,cHex_), 23.4 (*C*H_2,cHex_) ppm. MS (*m*/*z*): calcd C_32_H_47_N_3_O_15_NaPtS (M + Na)^+^, 963.23; found, 963.29. EA calcd C_32_H_47_N_3_O_15_PtS: C, 40.85; H, 5.03; N, 4.47; S, 3.41. Found: C, 40.82; H, 4.87; N, 4.16; S, 3.71.

#### (OC-6-34)-[(1*R*,2*R*)-1,2-cyclohexanediamino]-hydroxido-[(14-(4,7-dimethyl-1,3-dioxo-1,3,3a,4,7,7a-hexahydro-2*H*-4,7-epoxyisoindol-2-yl)-3,6,9,12-tetraoxatetradecyl)carbamato]oxalatoplatinum(iv) (Ox-OH-PMal)

A solution of DSC (85 mg, 0.331 mmol, 1.1 eq.) in anhydrous DMSO (1.4 mL) was added overnight to a stirring suspension of Ox-(OH)_2_ (140 mg, 0.327 mmol) in anhydrous DMSO (2.8 mL) under an argon atmosphere at RT in the dark with the aid of a syringe pump. After the final addition, the reaction mixture was stirred for an additional 1 h. PMal-PEG-NH_2_ (203 mg, 0.49 mmol, 1.5 eq.) was added and the reaction mixture was stirred for another 24 h at RT in the dark. DMSO was removed under high vacuum (50 °C), and the crude mixture was purified by preparative RP-HPLC (19% MeCN (+0.1% HCOOH) in H_2_O (+0.1% HCOOH); isocratic) and lyophilized to obtain the title compound as a white solid (163 mg, 57%). ^1^H NMR (500 MHz, DMSO-*d*_6_) *δ* 9.89–9.46 (m, 1H, N*H*_2_), 8.39–8.14 (m, 1H, N*H*_2_), 7.73–7.57 (m, 1H, N*H*_2_), 7.28–6.94 (m, 1H, N*H*_2_), 6.36 (s, 2H, =C*H*_Furane,*exo*_), 6.34–6.26 + 6.07–5.96 (m, 1H, N*H*_Ca_), 6.22 (s, 2H, =C*H*_Furane,*endo*_) 3.53–3.40 (m, 18H, C*H*_PEG_), 3.27 (s, 2H, C*H*_Furane,*endo*_), 3.10–3.00 (m, 2H, C*H*_2_NH_Ca_), 2.89 (s, 2H, C*H*_Furane,*exo*_), 2.58–2.47 (m, 2H, C*H*_cHex_), 2.14–2.00 (m, 2H, C*H*_cHex_), 1.62 (s, 6H, C*H*_3,Furane,*endo*_), 1.56–1.40 (m, 9H, C*H*_cHex_ + C*H*_3,Furane,*exo*_), 1.39–1.26 (m, 1H, C*H*_cHex_), 1.15–1.05 (m, 2H, C*H*_cHex_) ppm. MS (*m*/*z*): calcd C_29_H_46_N_4_O_14_NaPt (M + Na)^+^, 892.26; found, 892.32.

#### (OC-6-34)-[(1*R*,2*R*)-1,2-cyclohexanediamino]-oxalato-[4-(5,6-dimethoxybenzo[*b*]thiophen-2-yl)-4-oxobutylcarboxylato]-[(14-(2,5-dioxo-2,5-dihydro-1*H*-pyrrol-1-yl)-3,6,9,12-tetraoxatetradecyl)carbamato]platinum(iv) (Ox-MSA-Mal)

Ox-OH-PMal (58 mg, 0.067 mmol) was dissolved in DMF (10 mL) and stirred at 90 °C for 3 h in open air. The mixture was cooled down to RT, the solvent volume was reduced to *circa* 2–3 mL under high vacuum (50 °C) and the reaction mixture was left stirring under an argon atmosphere at RT. MSA-2 (40 mg, 0.13 mmol, 2 eq.) was dissolved in anhydrous DMF (1 mL) under an argon atmosphere at RT and TEA (28 μL, 0.2 mmol, 3 eq.) and TBTU (51 mg, 0.16 mmol, 2.5 eq.) were added and the reaction mixture was left stirring for 30 min at RT. The solution containing activated MSA-2 was added to the stirring solution of Ox-OH-Mal and the total reaction mixture was left stirring for 24 h at RT in the dark. DMF was removed under high vacuum (50 °C), and the crude mixture was purified by preparative RP-HPLC (31% MeCN (+0.1% CF_3_COOH) in H_2_O (+0.1% CF_3_COOH); isocratic) and lyophilized to afford the title compound as a white solid (48 mg, 68%). ^1^H NMR (500 MHz, DMSO-*d*_6_) *δ* 9.62–9.53 + 9.42–9.28 (m, 1H, N*H*_2_), 8.78–8.62 (m, 1H, N*H*_2_), 8.31–8.08 (m, 3H, N*H*_2_+ C*H*_Ar_), 7.60 (s, 1H, C*H*_Ar_), 7.47 (s, 1H, C*H*_Ar_), 7.02 (s, 2H, C*H*_Mal_), 6.78–6.72 + 6.34–6.23 (m, 1H, N*H*_Ca_), 3.86 (s, 3H, OC*H*_3_), 3.83 (s, 3H, OC*H*_3_), 3.58–3.53 (t, 2H, C*H̲*_2_N(CO)_2_), 3.53–3.49 (t, 2H, C*H*_PEG_), 3.48–3.43 (m, 12H, C*H*_PEG_), 3.37–3.26 (m, 3H, C*H*_2,MSA_ + C*H*_PEG_), 3.23–3.15 (m, 1H, C*H*_PEG_), 3.10–3.00 (m, 2H, C*H*_2_NH_Ca_), 2.75–2.53 (m, 2H, C*H*_2,MSA_ + C*H*_cHex_), 2.17–2.07 (m, 2H, C*H*_cHex_), 1.55–1.45 (m, 2H, C*H*_cHex_), 1.44–1.28 (m, 2H, C*H*_cHex_), 1.22–1.08 (m, 2H, C*H*_cHex_) ppm. ^13^C NMR (126 MHz, DMSO-*d*_6_) *δ* 192.8 (*C̲*OC_q,Ar_), 179.9 (PtO*C*O), 170.9 (2x*C*O_Mal_), 164.2 (*C*ONH_Ca_), 163.4 (*C*O_Ox_), 163.3 (*C*O_Ox_), 150.8 (*C*_q,Ar_), 148.5 (*C*_q,Ar_), 140.6 (*C*_q,Ar_), 135.7 (*C*_q,Ar_), 134.5 (2x*C*H_Mal_), 132.5 (*C*_q,Ar_), 130.3 (*C*H_Ar_), 106.5 (*C*H_Ar_), 104.3 (*C*H_Ar_), 69.7 (2x*C*H_2,PEG_), 69.6 (2x*C*H_2,PEG_), 69.5 (*C*H_2,PEG_), 69.4 (2x*C*H_2,PEG_), 69.2 (*C*H_2,PEG_), 66.9 (*C*H_2,PEG_), 60.9 (*C*H_cHex_), 60.7 (*C*H_cHex_), 55.8 (O*C*H_3,MSA_), 55.6 (O*C*H_3,MSA_), 40.7 (*C̲*H_2_NH_Ca_), 36.7 (*C̲*H_2_N(CO)_2_), 34.0 (*C*H_2,MSA_), 31.0 (*C*H_2,cHex_), 30.9 (*C*H_2,cHex_), 29.6 (*C*H_2,MSA_), 23.7 (*C*H_2,cHex_), 23.3 (*C*H_2,cHex_) ppm. MS (*m*/*z*): calcd C_37_H_50_N_4_O_17_NaPtS (M + Na)^+^, 1072.24; found, 1072.35. EA calcd C_37_H_50_N_4_O_17_PtS·1TFA: C, 40.24; H, 4.42; N, 4.81; S, 2.75. Found: C, 40.04; H, 4.41; N, 4.98; S, 2.88.

### Stability and reduction experiments

PB (150 mM, pH 7.4) containing 1 mM platinum compound was incubated at 20 °C, with and without the addition of 10 eq. of AA. The reaction was monitored using a Thermo Scientific Dionex UltiMate 3000 UHPLC system with a Waters Acquity UPLC BEH C18 1.7 μm 3.0 × 50 mm column. Milli-Q water containing 0.1% formic acid and acetonitrile containing 0.1% formic acid were used as eluents. A gradient of 5–95% over 5 min was used with a flow of 0.6 mL min^−1^. To evaluate the current state of the reaction, the peak area of the parental complex was used. This was done due to the fact that in most cases the reduction products did not have a sufficient retention time to be distinguished from the injection peak.

### SEC-ICP-MS measurements

FCS was purchased from Sigma-Aldrich and buffered with 150 mM PB pH 7.4 to guarantee a stable pH. The platinum(iv) complexes were dissolved in 150 mM PB (pH 7.4) at a concentration of 5 mM and diluted 1 : 100 in the buffered serum to obtain a final concentration of 50 μM. The samples were then incubated in an autosampler at 37 °C and analyzed for 24 h. Between each sample, a pure water blank was measured. For SEC-ICP-MS measurements an Agilent 1260 Infinity system coupled to an Agilent 7800 ICP-MS equipped with a dynamic reaction cell was used. Oxygen (purity 5.5, Messer Austria GmbH, Gumpoldskirchen, Austria) was used as a reaction gas. The parameters are given in Table S2.†

### Cell culture and preparation of drug stocks

The human monocytic THP1-Dual™ reporter cell model was purchased from InvivoGen (#thpd-nfis). The human colorectal cancer cell line HCT116 and the murine colorectal CT-26 cell model were purchased from ATCC (Manassas, VA, USA). The oxaliplatin-resistant subline HCT116/OxR was generated as published previously.^52^ All reagents were obtained from Sigma-Aldrich (Sigma-Aldrich, St Louis, MO, USA) unless stated otherwise. THP1-Dual cells were cultured in RPMI-1640 medium (R6504), supplemented with l-glutamine (2 mM), Normocin™ (InvivoGen), and Pen-Strep (100 U mL^−1^–100 μg mL^−1^). Cells were selected once-weekly by additionally supplementing Zeocin® (InvivoGen) and Blasticidin (InvivoGen). HCT116 and HCT116/OxR cells were cultured in McCoy's 5A modified medium (M8403) and CT-26 cells in Dulbecco's modified Eagle's medium/Ham's Nutrient Mixture F12 (DMEM/F12) (SAFC Biosciences Ltd, UK). All cell culture media were supplemented with 10% FCS (BioWest, Riverside, MO, USA). The cells were incubated under standard conditions (37 °C, 5% CO_2_), regularly checked for Mycoplasma contamination, and authenticated *via* short tandem repeat analysis (Microsynth, Vienna, Austria). Oxaliplatin (LCLabs; # O-7111) was prepared as 10 mM stock in water. SR-717 was prepared as 10 mM stock in dimethyl sulfoxide (DMSO; Sigma-Aldrich). MSA-2 was prepared as 10 mM stock dimethylformamide (DMF). The STING inhibitor H-151 was obtained from InvivoGen and was prepared as 7.8 mM DMSO stock. Ox-SR-PEG-Es was prepared as a 10 mM stock in DMSO, Ox-MSA-PEG-Es and Ox-MSA-PEG-Ca were prepared as a 100 mM DMF stock. AA was prepared freshly in a cell culture medium. All drug stocks were stored at −20 °C. Dilutions of stocks were prepared in a cell culture medium immediately before the experiment. Corresponding dilutions of DMF or DMSO were used as untreated vehicle controls.

### MTT-based cell viability assay

Cell models were seeded (2–2.5 × 10^3^ cells per well) in 96-well plates. After 24 h of recovery, the compounds were applied at the concentrations indicated in triplicate. Cell viability was determined after 72 h of continuous drug incubation using an MTT-based cell viability assay (EZ4U, Biomedica, Vienna, Austria) according to the manufacturer's recommendations. Absorbance was measured at 450 nm (*vs.* 620 nm as reference) using a multimode microplate reader (Tecan Spark, Zurich, Switzerland). Half-maximal inhibitory concentrations (IC_50_ values) were derived *via* interpolation from dose–response curves, modeled with the four-parameter logistic (4PL) nonlinear regression model in GraphPad Prism (La Jolla, CA, USA) from at least three independent experiments. Drug interaction dynamics between oxaliplatin and MSA-2 were established using the ZIP synergy scoring model of SynergyFinder (v3.14).^53^

### Western blot analysis

The THP1-Dual cell model was seeded at 1 × 10^6^ cells per well in six-well plates. After 24 h of recovery, the compounds were applied at the concentrations indicated. After 24 h of drug exposure, whole-cell protein isolation was performed as described previously.^14^ Sodium dodecyl sulfate polyacrylamide gel electrophoresis was performed. Proteins were transferred onto polyvinylidene difluoride membranes (Thermo Scientific, Waltham, MA, USA). Antibodies targeting STING (##13 647; 1 : 1000), phospho-STING (#19 781; 1 : 500), TBK1 (#3504; 1 : 500), IRF-3 (#4302; 1 : 500), phospho-p65 (#3033; 1 : 500), ISG15 (#2743; 1 : 1000), caspase-3 (#9665; 1 : 1000), cleaved caspase-3 (#9661; 1 : 1000), PARP (#9532; 1 : 1000), and cleaved PARP (#9541; 1 : 1000) were purchased from Cell Signaling Technology (Danvers, MA, USA). The p65 antibody (#sc-372; 1 : 1000) was purchased from Santa Cruz and the phospho-H2A.X antibody (#05-636-I; 1 : 500) was obtained from Merck (Darmstadt, Germany). The anti-β-actin (#5441; 1 : 2000) antibody was obtained from Sigma-Aldrich. The anti-mouse IgG (#A0168; 1 : 10 000) horseradish peroxidase (HRP)-coupled secondary antibody was purchased from Sigma-Aldrich, and the anti-rabbit IgG (#7074; 1 : 5000) HRP-linked secondary antibody was obtained from Cell Signaling Technology.

### Intracellular platinum accumulation *in vitro* by ICP-MS

The THP1-Dual cell model was seeded at 1 × 10^6^ cells per well in six-well plates. After 24 h of recovery, the compounds were applied at the concentrations indicated in triplicate. After 3 h of drug exposure, the cells were prepared as published previously.^13^ In brief, the cells were detached, washed twice in Dulbecco's Phosphate-Buffered Saline (DPBS) (Cytiva), dried at RT overnight and lysed in 400 μL of nitric acid (68% Rotipuran Supra, Carl Roth, Karlsruhe, Germany) for 2 h at RT. The lysate was filled up to 8 mL with water. The platinum concentration was determined by ICP-MS analysis. Platinum and rhenium standards were derived from CPI International (Amsterdam, the Netherlands). The total platinum content was determined with an Agilent 7800 quadrupole-based ICP-MS instrument (Agilent Technologies, Tokyo, Japan) equipped with an Agilent SPS 4 autosampler (Agilent Technologies, Tokyo, Japan) and a MicroMist nebulizer at a sample uptake rate of approximately 0.2 mL min^−1^. A radio frequency power of 1550 W was used as well as nickel cones. Argon was used as a plasma gas (15 L min^−1^) and as a carrier gas (∼1.1 L min^−1^). The dwell time was set to 0.1 s and the measurements were performed in 12 replicates with 100 sweeps. Rhenium served as an internal standard for platinum. The Agilent MassHunter software package (Workstation Software, version C.01.04, 2018) was used for data processing.

### IRF and NF-κB-inducible THP1-dual reporter cell model

The THP1-Dual™ reporter cell model for simultaneously monitoring the activity of the IRF and NF-κB pathway contains an IRF-inducible luciferase reporter under the control of an ISG54 minimal promoter in conjunction with five interferon (IFN)-stimulated response elements, and an induced SEAP driven by an IFN-β minimal promoter fused to five copies of the NF-κB consensus transcriptional response element and three copies of the c-Rel binding site. The luciferase and SEAP reporter are measurable in the cell culture supernatant using QUANTI-Luc™ 4 Lucia/Gaussia (InvivoGen) and QUANTI-Blue™ (InvivoGen) solution, respectively. Cells were seeded at 1 × 10^5^ cells per well in 96-well plates in RPMI-1640 medium, supplemented with 10% FCS, l-glutamine and Pen-Strep. After 2 h of recovery, the compounds were applied at the concentrations indicated in triplicate. The cell culture supernatant (SN) was collected after 24 h of drug incubation and secreted luciferase and SEAP reporter levels were measured according to the manufacturer's recommendations. In brief, for the detection of luciferase, 20 μL of SN were transferred into 96-well white opaque plates, and 30 μL of QUANTI-Luc™ reagent were added per well using an injector module in a multimode microplate reader (Tecan Spark, Zurich, Switzerland). Luminescence was recorded at a reading time of 0.1 second. For the detection of SEAP, 10 μL of SN were transferred to 96-well plates and 90 μL of QUANTI-Blue™ were added per well. Absorbance was measured at 620 nm. Half-maximal effective concentrations (EC_50_ values) were derived *via* interpolation from dose–response curves, modeled with the four-parameter logistic (4PL) nonlinear regression model in GraphPad Prism. For comparison, the maximal response rate of the prodrug was depicted in the percent of the maximal response rate achieved by SR-717 or MSA-2 (%*E*_MAX_) at the respective concentrations tested. Activatability, tested in co-incubation experiments with AA (50 μM), was expressed as the area under the curve (AUC) ratio of +AA *vs.* −AA-treated cells. The STING-dependency of the reporter induction was tested by co-administration of the STING inhibitor H-151 (10 μM), added to the cell culture medium 2 h before applying the investigated compounds. In addition to monitoring secreted reporter levels, the cell viability of the reporter cells was determined after 24 h of drug treatment by an MTT-based cell viability assay.

### Quantitative RT-PCR

The THP1-Dual cell model was seeded at 1 × 10^6^ cells per well in six-well plates. After 24 h of recovery, the compounds were applied at the concentrations indicated. After 24 h of drug exposure, total RNA was isolated using TRIzol reagent (Thermo Fisher Scientific, MA, USA), as described previously.^12^ RNA was reverse transcribed into cDNA using RevertAid Reverse Transcriptase (Thermo Fisher Scientific). Real-time PCR was performed using the GoTaq® RT-qPCR system (Promega) with a CFX96 Touch Real-Time PCR Detection System (Bio-Rad Laboratories). Relative mRNA levels were quantified using the 2^−ddCT^ method.^54^ RPL41 was used as a housekeeping gene. Human-specific primers were purchased from Eurofins Genomics (Ebersberg, Germany) with the following sequences: RPL41: fwd (5′-CAAGTGGAGGAAGAAGCGA-3′), rev (5′-TTACTTGGACCTCTGCCTC-3′); IFNB1: fwd (5′-CTT GGA TTC CTA CAA AGA AGC AGC-3′), rev (5′-TCCTCCTTCTGGAACTGCTGCA-3′); ISG15: fwd (5′-GAGAGGCAGCGAACTCATCT-3′), rev (5′-CTTCAGCTCTGACACCGACA-3′); IFIT1: fwd (5′-GCCTTGCTGAAGTGTGGAGGAA-3′), rev (5′-ATCCAGGCGATAGGCAGAGATC-3′); CXCL10: fwd (5′-GGTGAGAAGAGATGTCTGAATCC-3′), rev (5′-GTCCATCCTTGGAAGCACTGCA-3′); and CCL5: fwd (5′-ATCCTCATTGCTACTGCCCTC-3′), rev (5′-GCCACTGGTGTAGAAATACTCC-3′).

### Cytokine and chemokine detection using a Luminex xMAP detection platform

Quantification of secreted protein targets in either cell culture SN or in plasma of treated animals was performed using a ProcartaPlex (96-well) multiplex assay panel (Thermo Fisher Scientific) in the xMAP detection platform (Luminex). For the *in vitro* detection of secreted proteins, THP1-Dual cells were seeded at 1 × 10^5^ cells per well in 96-well plates in RPMI-1640 medium, supplemented with 10% FCS, l-glutamine and Pen-Strep. After 24 h of recovery, the compounds were applied at the concentrations indicated in triplicate.

After 24 h of drug incubation, the cell culture SN of technical triplicate was collected, pooled, centrifuged (400*g*, 10 min, 4 °C), transferred into a fresh tube, and stored at −20 °C.

For protein detection *in vivo*, mouse blood was collected from the submandibular vein in K3E K3EDTA-tubes (Greiner Bio-One; Kremsmünster, AUT; #450 530). Following analysis of hematological parameters (see also “*In vivo* syngraft experiments” in the Experimental section), EDTA-blood was centrifuged (900*g*, 10 min, 4 °C) twice and the supernatant (EDTA-plasma) was stored at −20 °C. Next, the ProcartaPlex (96-well) multiplex assay panel was performed according to the manufacturer's recommendations. In brief, the capture bead mix was incubated with test samples (undiluted SN or mouse plasma) or standard mix overnight. The SN of three independent experiments or the plasma of four animals per treatment group was loaded onto the multiplex assay plate. Next, biotinylated detection antibody mix and Streptavidin-PE were added to the plate. Following the recommended washing steps, the samples were incubated in a reading buffer and were measured using a Luminex xMAP platform (Bio-Plex MAGPIX Multiplex Reader; Bio-Rad Laboratories) and analyzed using xPONENT software.

### *In vivo* syngraft experiments

Mice were kept in groups of four per cage with sterilized paper-based environmental enrichment and food and water *ad libitum*, under pathogen-free conditions and a controlled environment with a 12 h light–dark cycle. Syngeneic models of colorectal carcinoma were generated *via* subcutaneous injection of CT-26 cells (0.5 × 10^6^ cells in 50 μL of serum-free RPMI-1640 medium) into the right flank of 8–12 weeks old BALB/c mice. Oxaliplatin was dissolved in 5% glucose and Ox-MSA-PEG-Ca, Ox-MSA-Mal, and MSA-2 in 15% PEG400 in DPBS (Cytiva). Ox-MSA-PEG-Ca (21.52 mg kg^−1^), Ox-MSA-Mal (26.37 mg kg^−1^), and MSA-2 (6.67 mg kg^−1^) were administered equimolar to the MTD of oxaliplatin (=9 mg kg^−1^). For the drug distribution study by ICP-MS and immune phenotyping by flow cytometry, animals were treated 6 d after subcutaneous implantation, when tumors reached a volume of ∼140 mm^3^, intravenously (i.v.) with the respective compounds or solvent twice a week for one week. All animals were euthanized 24 h after the second dose. For examining hematotoxic effects after long-term treatment, C57BL/6 mice were treated i.v. with the relevant compounds or solvent, twice a week for two weeks and full blood was analyzed 72 h after the fourth dose. To determine the anticancer efficacy of the investigated compounds, treatment started 4 d after tumor implantation, when all tumors were measurable (>25 mm^3^) using a twice-weekly treatment scheme for two weeks. Tumor size was determined daily *via* manual caliper measurement. The tumor volume was calculated as tumor volume = (length × diameter × diameter)/2. The overall condition, shape, and activity of the animals were assessed daily. Peripheral blood was collected from the submandibular vein in K3E K3EDTA tubes (Greiner Bio-One; Kremsmünster, AUT) on ice and was analyzed for hematological parameters (red blood cells (RBCs), hemoglobin (HGB), hematocrit (HCT), red cell distribution width (RDW), mean corpuscular volume (MCV), white blood cells (WBCs), lymphocytes (LYM), monocytes (MO), granulocytes (GRA), and platelets (PLT) using a Vet ABC hematology analyzer (scil Animal Care GmbH, Viernheim, Germany). EDTA-blood was centrifuged (900*g*, 10 min) twice and the supernatant (EDTA-plasma) was stored at −20 °C for subsequent cytokine/chemokine analysis using a Luminex detection platform. All animal experiments were performed with humane endpoints based on Federation of European Laboratory Animal Science Associations guidelines and according to the regulations of the Ethics Committee for the Care and Use of Laboratory Animals at the Medical University Vienna (proposal number BMBWF 2023–0.122.324). Animals were euthanized upon reaching a tumor volume of >1500 mm^3^, a loss in body weight (>20%), tumor ulceration or other indications of deteriorated health. Animals were euthanized *via* cervical dislocation.

### Platinum distribution study *in vivo* by ICP-MS

The generation and treatment of tumor-bearing mice are outlined in the section “*In vivo* syngraft experiments” described above. Mouse tissues were processed as described previously.^13^ Briefly, serum was isolated from peripheral blood by centrifugation (900*g*, 10 min, 4 °C, twice). Tissues were digested in 20% nitric acid and hydrogen peroxide using an open-vessel graphite digestion system. The platinum concentration was determined by ICP-MS analysis; for details see “Intracellular platinum accumulation *in vitro* by ICP-MS”.

### Immune activation and infiltration by flow cytometry

The generation and treatment of tumor-bearing mice are outlined in the section “*In vivo* syngraft experiments” above. Samples were kept on ice during the whole procedure. Tissues were prepared as described previously.^55^ In brief, tumor and spleen tissues were collected and mechanically dissociated in DPBS buffer containing Ca^2+^/Mg^2+^ and FCS (5%). Tumor samples were digested using collagenase VIII (1 mg mL^−1^) in a DPBS buffer (without Ca^2+^/Mg^2+^) supplemented with DNAse I (1 mg mL^−1^) for 15 min at 37 °C under constant shaking. Samples were filtered using 70 μm cell strainers in a DPBS buffer (without Ca^2+^/Mg^2+^) containing FCS (5%), EDTA (5 mM) and DNase I (20 μg mL^−1^). Subsequent washing steps were performed in DPBS buffer (without Ca^2+^/Mg^2+^) containing FCS (5%), EDTA (5 mM) and DNase I (20 μg mL^−1^), unless indicated otherwise. Red blood cells were removed by short incubation with ACK buffer. For blocking, samples were incubated with the Ultra-LEAF purified anti-mouse CD16/CD32 antibody (clone: 93; #101330; RRID:AB_2783037; dilution: 1 : 1000) (Biolegend, San Diego, CA, USA) in the BD Horizon Brilliant Stain Buffer (#563794; BD Biosciences) for 10 min. Next, the samples were incubated with an antibody mixture in the BD Horizon Brilliant Stain Buffer for 30 min. Fluorescent dye-labeled antibodies targeting murine CD3 (17A2; #100203; RRID:AB_312661; 1 : 50), CD8a (53-6.7; #100738; RRID:AB_11204079; 1 : 160), CD19 (6D5; #115528; RRID:AB_493735; 1 : 400), CD49b (DX5; #108924; RRID:AB_2565271; 1 : 229), CD11b (M1/70; #101228; RRID:AB_893232; 1 : 80), and CD69 (H1.2F3; # 104512; RRID:AB_493564; 1 : 40) were purchased from BioLegend. The antibody targeting murine CD4 (RM4-5; # 85-0042-U100; 1 : 80) and the viability dye Ghost Dye Red 780 (#13-0865-T100; 1 : 1000) were purchased from Tonbobio (San Diego, CA, USA). The antibody targeting CD45 (30-F11; #363-0451-82; RRID:AB_2925264; 1 : 80) was purchased from Life Tech (Invitrogen). For intracellular staining of granzyme B (GzmB) (QA16A02; #372208; RRID:AB_2687032; 1 : 20) and FoxP3 (MF-14; #320014; RRID:AB_439750; 1 : 100) the True-Nuclear Transcription Factor Buffer Set (Biolegend; #424401) was used according to the manufacturer's recommendations. In brief, samples were incubated with Perm Fix Solution for 60 min at RT. Next, the samples were stained with antibodies targeting GzmB and FoxP3 (diluted in Perm buffer) for 30 min at RT. Then, the samples were resuspended in DPBS buffer containing FCS (5%), EDTA (5 mM) and DNase I (20 μg mL^−1^) and incubated overnight at 4 °C. Next day, the samples were analyzed using a Cytek Aurora 5L (Cytek Biosciences, CA, USA) spectral flow cytometer. Data were analyzed using SpectroFlo (v3.2.1) (Cytek Biosciences) and FlowJo (v10.8.1) (FlowJo LLC, Ashland, OR, USA) software.

### Data evaluation and statistical analysis

The statistical tests used to determine significance and the corresponding *p* values are described in the respective figure legends. Significance was set at *p* < 0.05. All Student's *t*-tests are unpaired and two-sided unless indicated otherwise. One-way analysis of variance (ANOVA) was performed with the Tukey multiple comparison test unless indicated otherwise. Tumor sizes were compared using two-way ANOVA with Tukey's multiple comparison test. Statistical tests were conducted using GraphPad Prism version 9.3.0 for Windows (GraphPad Software Inc., San Diego, CA, USA). Error bars represent the standard deviation unless indicated otherwise.

## Author contributions

Conceptualization: CRK and WB; data curation: MD, MG, and AF; formal analysis: MD, MG, and AF; funding acquisition: CRK and WB; investigation: MD, MG, AF, MaG, CJ, and JVB; methodology: MD, MG, AF, MaG, and CJ; project administration: WB and CRK; resources: CRK and WB; supervision: CRK and WB; validation: MD, MG, AF, CRK, WB, MaG, and JVB; visualization: MD, MG, AF CRK, WB, CP, and DV; roles/writing – original draft: MD, MG, CRK, WB, MaG, CP, and DV; and writing – review & editing: MD, MG, MaG, AF, CJ, JVB, PV, CP, DV, PH, CRK, and WB.

## Conflicts of interest

The authors declare no competing financial interest.

## Supplementary Material

QI-012-D5QI00433K-s001

## Data Availability

The data supporting this article have been included as part of the ESI.†
