# Supplementary material for: Albumin-targeted oxaliplatin(iv) prodrugs bearing STING agonists
Source: Inorg Chem Front. 2025 Apr 4;12(13):4284–305. doi: 10.1039/d5qi00433k (PMC11969438; doi:10.1039/d5qi00433k)
Supplement: QI-012-D5QI00433K-s001 [file QI-012-D5QI00433K-s001.pdf]

Supplementary Information (SI) for Inorganic Chemistry Frontiers.
This journal is © the Partner Organisations 2025

**UPDATED 13 May 2025. The original version of this SI was first published on 04 April 2025. This version of the SI replaces the previous version in which Figure S3 was not shown correctly.**

# Albumin-Targeted Oxaliplatin(IV) Prodrugs Bearing STING Agonists

Martijn Dijkstra,‡[a,b] Michael Gutmann,‡[c] Mathias Gradl,[c] Anja Federa,[a,b] Carola Jaunecker,[c] John Vasco Breitenstein,[c] Petra Vician,[c] Christine Pirker,[c] Daniel Valcanover,[c] Petra Heffeter,[c,d] Bernhard K. Keppler,[a,d] Walter Berger,[c,d*] and Christian R. Kowol[a,d*]

A) University of Vienna, Faculty of Chemistry, Institute of Inorganic Chemistry, Waehringer Str. 42, 1090 Vienna, Austria. B) University of Vienna, Vienna Doctoral School in Chemistry (DoSChem), Waehringer Str. 42, 1090 Vienna, Austria. C) Center for Cancer Research and Comprehensive Cancer Center, Medical University of Vienna, Borschkegasse 8a, 1090 Vienna, Austria. D) Research Cluster "Translational Cancer Therapy Research", 1090 Vienna, Austria.

*Email: christian.kowol@univie.ac.at;

*Email: walter.berger@meduniwien.ac.at

‡These authors contributed equally to this work; *shared last author position.

## Table of contents


| | Content | Page |
|---|---|---|
| Figure S1 | Reduction kinetics of **Ox-Bz**, **Ox-Bz-F$_2$**, **Ox-Bz-F$_2$-NH$_2$** and **Ox-Bz-F$_2$-NHAc** | 2 |
| Table S1 | Mean IC$_{50}$ values (± SD) | 3 |
| Figure S2 | IC$_{50}$ ratios between HCT116 and HCT116/OxR | 4 |
| Figure S3 | Cytotoxic activity of SR-717 and MSA-2 alone or in combination with oxaliplatin | 5 |
| Figure S4 | Western blot analysis of STING and downstream components | 6 |
| Figure S5 | IRF and NF-κB pathway induction by oxaliplatin | 6 |
| Figure S6 | Impact of STING inhibition (H-151) on IRF and NF-κB pathway induction | 7 |
| Figure S7 | Western blot analysis of STING and downstream pathway activation in THP1-Dual | 8 |
| Figure S8 | mRNA expression levels | 9 |
| Figure S9 | Cytokine and chemokine secretion *in vitro* | 9 |
| Figure S10 | Sulfur traces of FCS | 10 |
| Figure S11 | SEC-ICP-MS results of **Ox-MSA-PEG-Ca** | 10 |
| Table S2 | SEC-ICP-MS operation parameters | 11 |
| Figure S12 | Immune cell activation and tumor infiltration | 11 |
| Figure S13 | Cytokine and chemokine secretion *in vivo* | 12 |
| Figure S14 | Flow cytometric gating strategy | 13 |
| Figure S15 | Long-term hematological effects | 14 |
| Figure S16 | Body weights of treated animals | 14 |
| Figure S17-S20 | $^1$H and DEPT $^{13}$C-NMR spectra and UHPLC chromatograms of final platinum(IV) complexes | 15-18 |

**UPDATED 13 May 2025. The original version of this SI was first published on 04 April 2025. This version of the SI replaces the previous version in which Figure S3 was not shown correctly.**

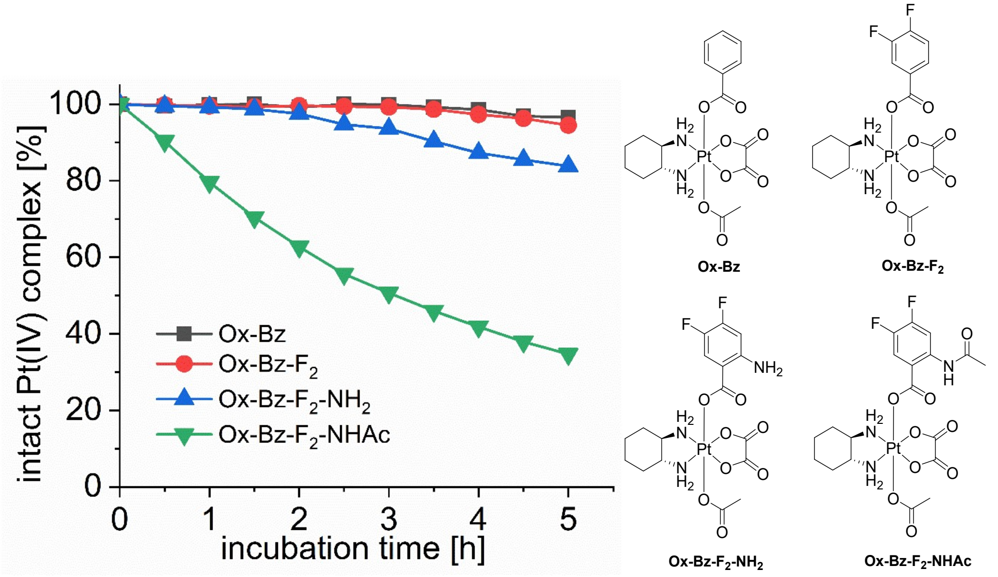


**Figure S1.** Reduction kinetics of 1 mM **Ox-Bz, Ox-Bz-F, Ox-Bz-F$_2$-NH$_2$** and **Ox-Bz-F$_2$-NHAc** in 150 mM phosphate buffer (pH = 7.4) at 20 °C with 10 eq. of L-ascorbic acid (AA) over 5 h, measured with UHPLC**.**

**UPDATED 13 May 2025. The original version of this SI was first published on 04 April 2025. This version of the SI replaces the previous version in which Figure S3 was not shown correctly.**

**Table S1.** Mean $IC_{50}$ values (± SD) of the investigated platinum(IV) prodrugs and oxaliplatin in various cancer cell models and impact AA (50 µM). $IC_{50}$ values were derived by interpolation from dose-response curves, modelled by four parameter logistic (4PL) non-linear regression after 72 h drug exposure.

| mean $IC_{50}$ (µM) ± *SD* | | | THP1-Dual | HCT116 | HCT116/OxR | CT26 |
|---|---|---|---|---|---|---|
| **oxaliplatin** | **- AA** | **$IC_{50}$** | 1.1 | 0.8 | 27.5 | 1.8 |
| | | **± *SD*** | *0.2* | *0.1* | *5.1* | *0.6* |
| | **+ AA** | **$IC_{50}$** | 0.8 | 0.8 | 25.1 | 2.0 |
| | | **± *SD*** | *0.1* | *0.1* | *6.3* | *0.3* |
| **Ox-SR-PEG-Es** | **- AA** | **$IC_{50}$** | 2.3 | 3.2 | 64.5 | 19.6 |
| | | **± *SD*** | *0.8* | *1.1* | *4.7* | *3.1* |
| | **+ AA** | **$IC_{50}$** | 0.9 | 1.57 | 32.32 | 5.04 |
| | | **± *SD*** | *0.0* | *0.3* | *6.1* | *0.5* |
| **Ox-MSA-PEG-Es** | **- AA** | **$IC_{50}$** | 9.4 | 32.5 | > 100 | 42.4 |
| | | **± *SD*** | *0.8* | *7.7* | - | *1.4* |
| | **+ AA** | **$IC_{50}$** | 5.9 | 17.4 | 92.5 | 22.0 |
| | | **± *SD*** | *1.3* | *1.1* | *6.5* | *2.2* |
| **Ox-MSA-PEG-Ca** | **- AA** | **$IC_{50}$** | 11.9 | 32.0 | 85.0 | 53.8 |
| | | **± *SD*** | *3.6* | *6.0* | *13.0* | *16.1* |
| | **+ AA** | **$IC_{50}$** | 7.6 | 18.9 | 80.1 | 21.5 |
| | | **± *SD*** | *0.6* | *1.9* | *12.6* | *3.1* |

**UPDATED 13 May 2025. The original version of this SI was first published on 04 April 2025. This version of the SI replaces the previous version in which Figure S3 was not shown correctly.**

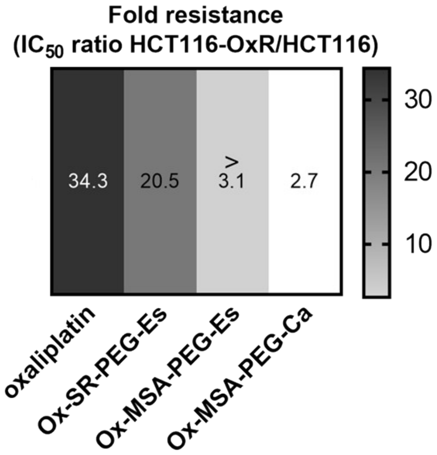


**Figure S2.** $IC_{50}$ ratios of the investigated drugs between parental HCT116 and an oxaliplatin-resistant subline HCT116/OxR**.** $IC_{50}$ values were derived by interpolation from dose-response curves, modelled by 4PL non-linear regression after 72 h drug exposure of HCT116 and HCT116/OxR cells. The $IC_{50}$ ratio was calculated for each compound between the parental and resistant cell model. Data are derived from at least three independent experiments in triplicate.

**UPDATED 13 May 2025. The original version of this SI was first published on 04 April 2025. This version of the SI replaces the previous version in which Figure S3 was not shown correctly.**

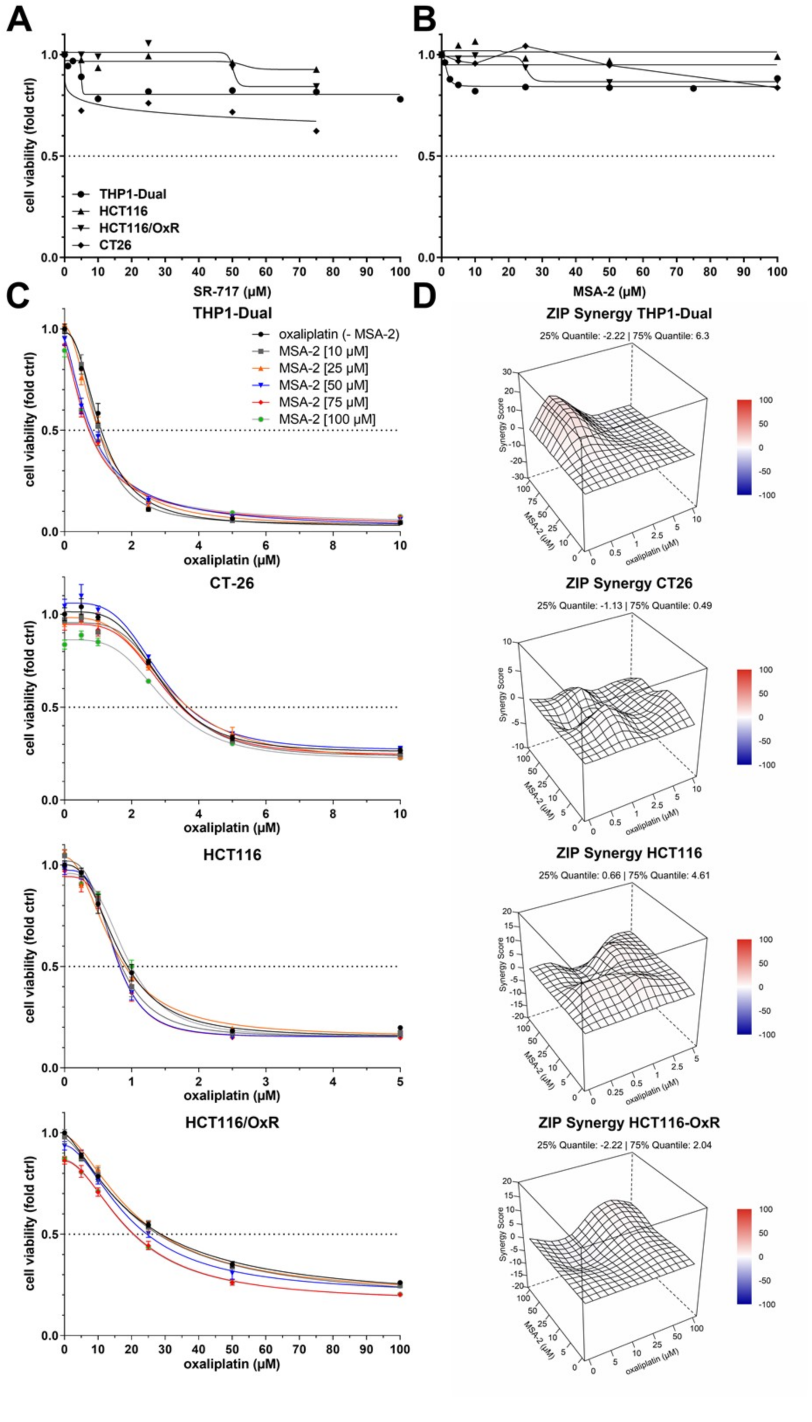


**Figure S3.** Cytotoxic activity of SR-717 and MSA-2 alone or in combination with oxaliplatin. Impact of 72 h continuous drug exposure on the viability of the indicated cancer cell lines, determined by an MTT-based assay. Dose-response curves, modelled by 4PL non-linear regression of single agent SR-717 (A), MSA-2 (B), and combinations of MSA-2 with oxaliplatin (C). The ZIP synergy scoring model (SynergyFinder) was used to calculate the degree of interaction between oxaliplatin and MSA-2 (D). One representative experiment out of at least three performed is shown. ctrl = control.

**UPDATED 13 May 2025. The original version of this SI was first published on 04 April 2025. This version of the SI replaces the previous version in which Figure S3 was not shown correctly.**

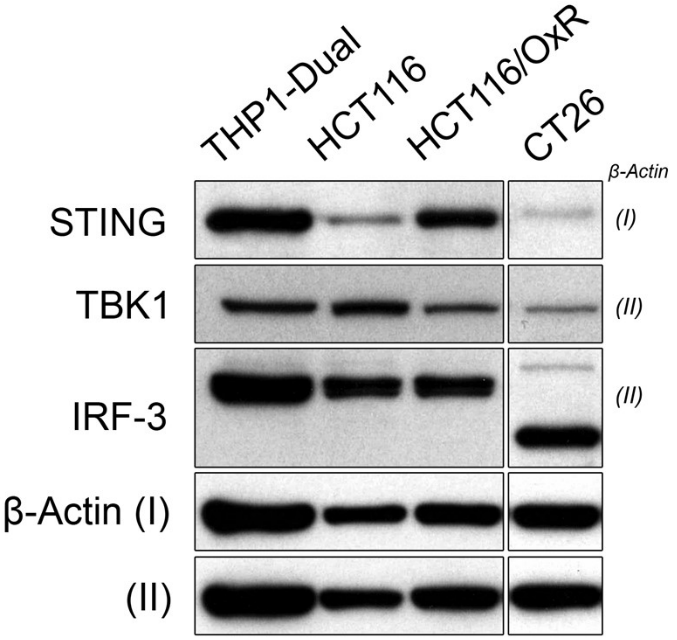


**Figure S4.** Western blot analysis of protein expression levels of STING and downstream components in cancer cell models of human (THP1-Dual, HCT116 and HCT116/OxR cells) and murine (CT-26) origin.

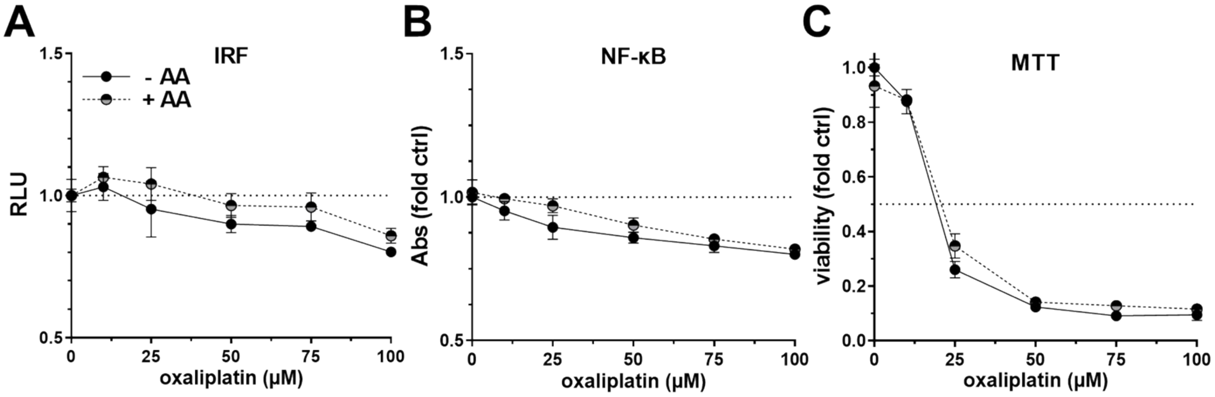


**Figure S5.** IRF and NF-κB pathway induction by oxaliplatin and impact of reducing conditions in the THP1-Dual reporter cell model. IRF-inducible luciferase (A), as well as NF-κB-inducible SEAP reporter levels (B) were measured in the THP1-Dual cell culture supernatant following 24 h continuous exposure to oxaliplatin. Impact of reducing conditions were tested by co-incubation with AA (50 µM). Impact of oxaliplatin on viability of the reporter cell model was determined by an MTT-based assay in C. One representative experiment out of at least three performed is shown. Data points in A-C are shown as mean ± SD. IRF = Interferon regulatory factor; NF-κB = Nuclear factor kappa-light-chain-enhancer of activated B cells; MTT = 3-(4,5-dimethylthiazol-2-yl)-2,5-diphenyltetrazolium bromide; RLU = relative light units; Abs = absorbance; ctrl = control; AA = L-ascorbic acid. *$p < 0.05$; **$p < 0.01$; ***$p < 0.001$.

**UPDATED 13 May 2025. The original version of this SI was first published on 04 April 2025. This version of the SI replaces the previous version in which Figure S3 was not shown correctly.**

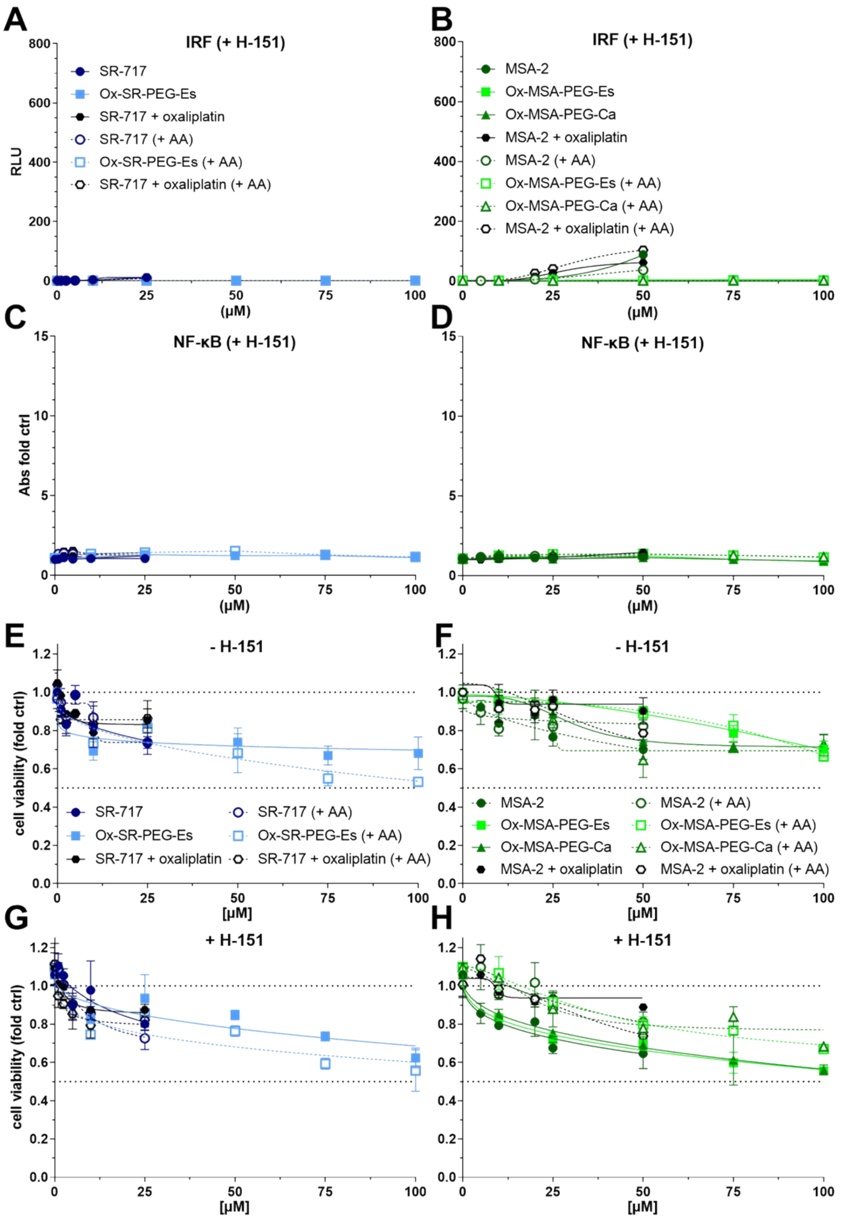


**Figure S6.** Impact of STING inhibition (H-151) on IRF and NF-κB pathway induction by Pt(IV)-prodrugs as compared to reference compounds in the THP1-Dual reporter cell model, with cell viability used for normalization. The STING inhibitor H-151 (10 μM) was incubated for 2 h before cells received the indicated drugs or combinations. Oxaliplatin was always applied at 5 μM. IRF-inducible luciferase (A, B), as well as NF-κB-inducible SEAP reporter levels (C, D) were measured in the THP1-Dual cell culture supernatant following 24 h continuous exposure to the indicated drugs or the combinations. (E-H) MTT-based cell viability curves used for normalization of the data shown in Figure 5. Dose-response curves were modelled by 4PL non-linear regression model. One representative experiment out of at least three performed is shown. Data points are shown as mean ± SD. IRF = Interferon regulatory factor; NF-κB = Nuclear factor

**UPDATED 13 May 2025. The original version of this SI was first published on 04 April 2025. This version of the SI replaces the previous version in which Figure S3 was not shown correctly.**

kappa-light-chain-enhancer of activated B cells; RLU = relative light units; Abs = absorbance; ctrl = control; AA = L-ascorbic acid. *$p < 0.05$; **$p < 0.01$; ***$p < 0.001$.

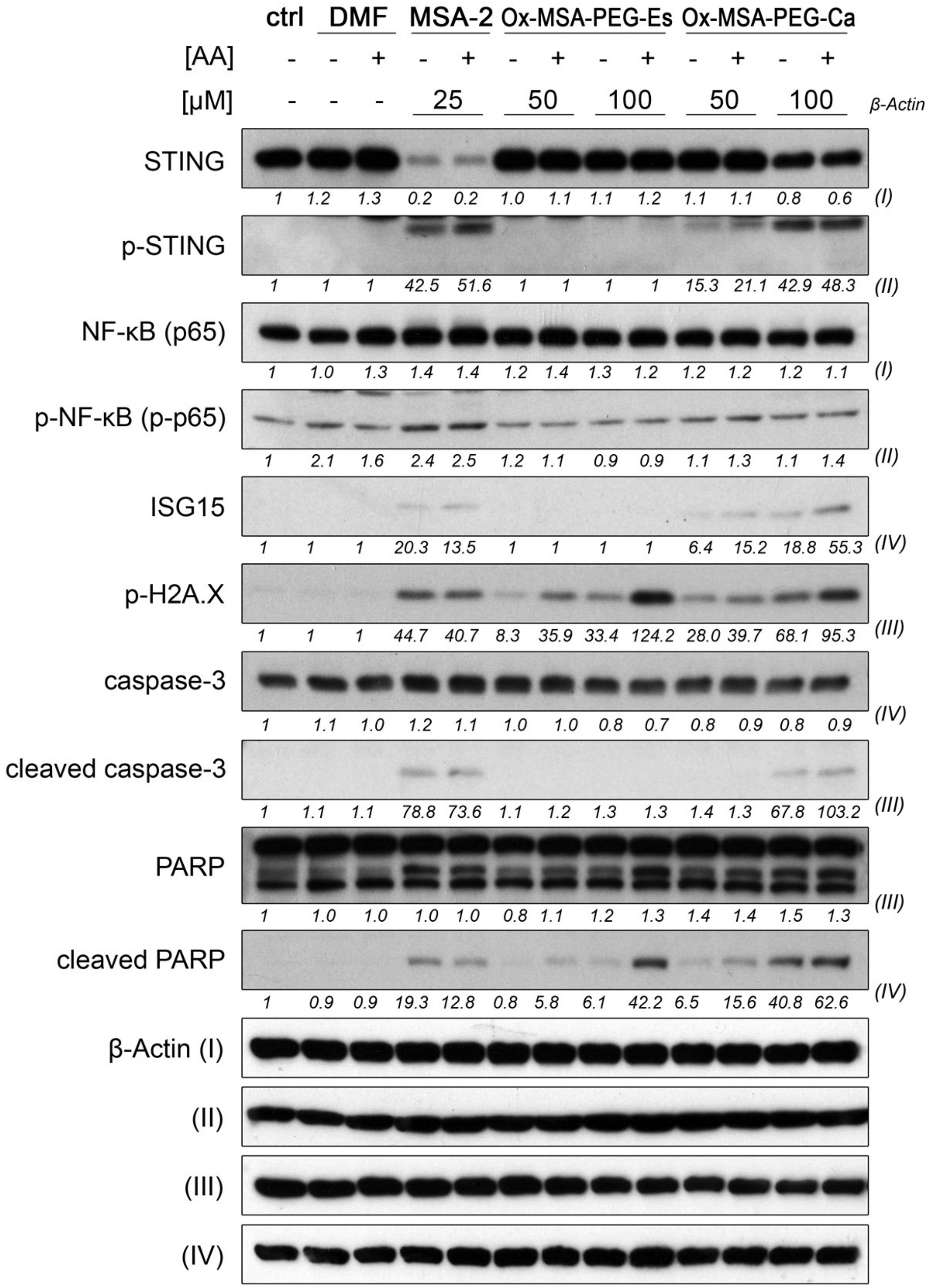


**Figure S7.** Western blot analysis of STING and downstream pathway activation in THP1-Dual cells. Protein expression levels were analyzed following 24 h continuous drug exposure in the THP1-Dual cell model under ± reducing conditions (50 μM AA). ctrl = control; AA = L-ascorbic acid.

**UPDATED 13 May 2025. The original version of this SI was first published on 04 April 2025. This version of the SI replaces the previous version in which Figure S3 was not shown correctly.**

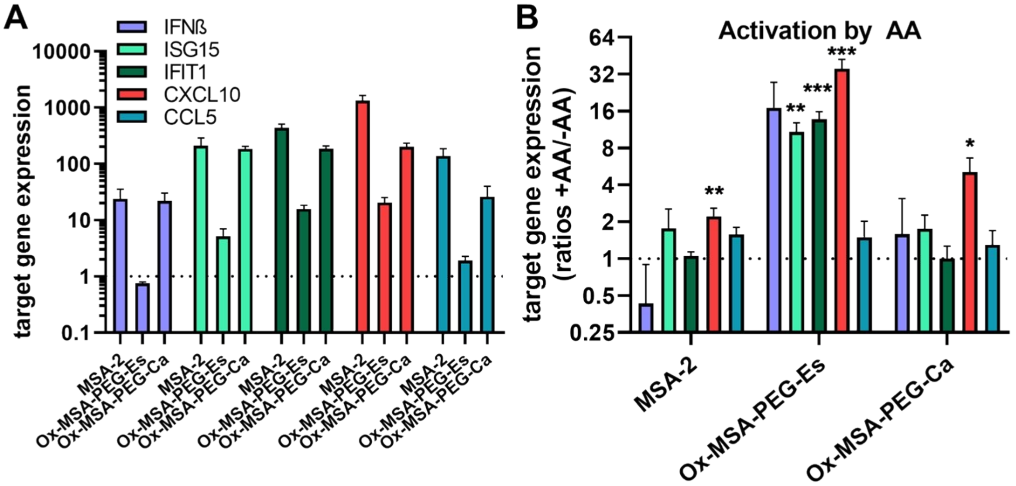


**Figure S8**. mRNA expression levels of targets downstream of STING. mRNA expression levels were determined following 24 h continuous drug exposure with MSA-2-relasing platinum(IV)-prodrugs (100 µM) or MSA-2 (25 µM) in the THP1-Dual cell model under ± reducing conditions (50 µM AA) by quantitative RT-PCR. Data points are shown as mean ± SD of normalized target gene expression (A) or as the ratio of the normalized target gene expression between +AA and -AA. The statistical significance of differences in target gene expression ratios between +AA and -AA (B) was tested by Student's t-test. AA = L-ascorbic acid. *p < 0.05; **p < 0.01; ***p < 0.001.

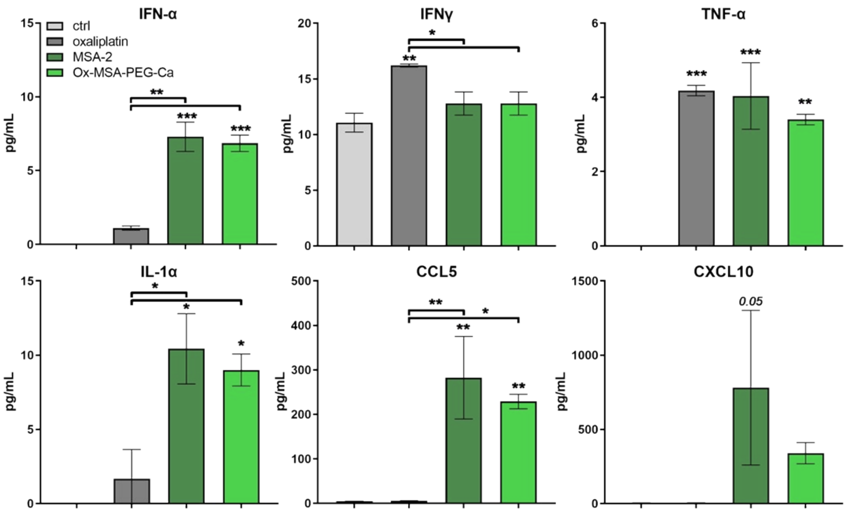


**Figure S9.** Cytokine and chemokine secretion in the THP1-Dual reporter cell model following exposure to a MSA-2-releasing platinum(IV)-prodrug (100 µM) as compared to oxaliplatin (5 µM) and MSA-2 (25 µM). Cell culture supernatants of THP1-Dual cells, exposed to the indicated drugs for 24 h, were analyzed for secreted cytokines and chemokines by a Luminex detection platform. Data points are shown as mean ±

**UPDATED 13 May 2025. The original version of this SI was first published on 04 April 2025. This version of the SI replaces the previous version in which Figure S3 was not shown correctly.**

SD. Data are derived from three independent experiments in triplicate. The statistical significance of differences in cytokine/chemokine levels between the treatment groups was tested using one-way-ANOVA (with the Tukey multiple comparison test). *$p < 0.05$; **$p < 0.01$; ***$p < 0.001$.

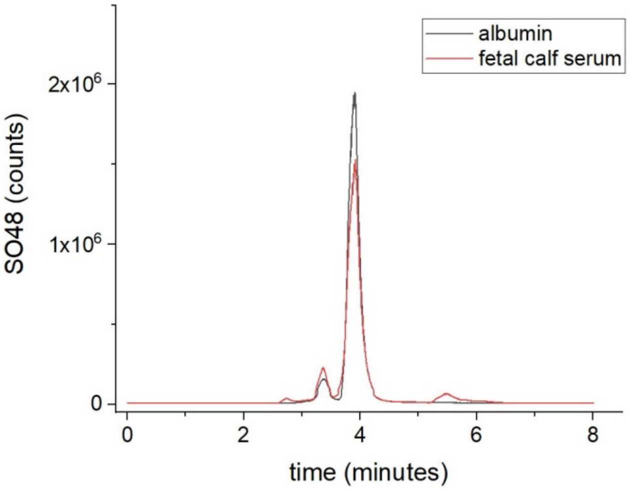


**Figure S10**. $^{48}$SO sulfur traces of FCS (+150 mM PB, pH 7.4) as well as pure albumin in PB (50 mM, pH 7.4), both measured by SEC-ICP-MS. The small peak at ~3.3 min corresponds to the albumin dimer.

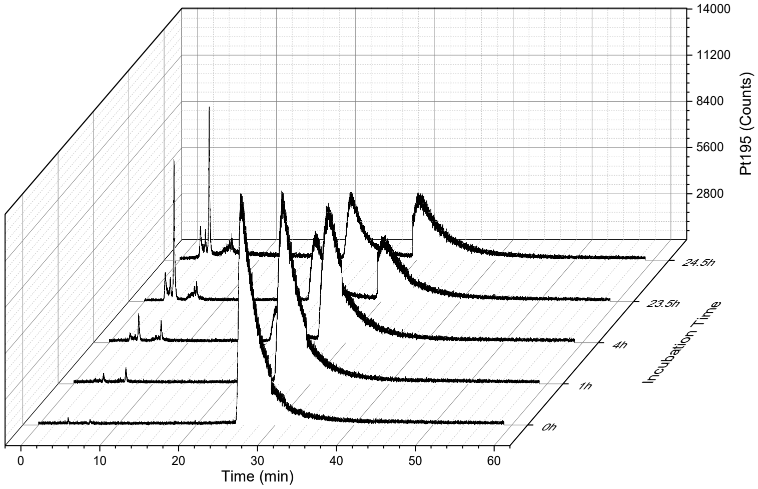


**Figure S11**. $^{195}$Pt-traces of incubation of 50 µM **Ox-MSA-PEG-Ca** in FCS (containing 150 mM phosphate buffer, pH = 7.4) at 37°C over 24 h, measured with SEC-ICP-MS.

**UPDATED 13 May 2025. The original version of this SI was first published on 04 April 2025. This version of the SI replaces the previous version in which Figure S3 was not shown correctly.**

**Table S2**. Operation parameters used in SEC-ICP-MS measurements

| | |
|---|---|
| Samples | 50 µM in FCS (150 mM PB, pH 7.4) |
| Column | Acquity UPLC BEH 200Å 1.7 µm, 4.6x150 mm |
| Eluent | 50 mM $CH_3COONH_4$, pH = 6.8 |
| Flow rate | 400 µl/min |
| Column temperature | 37°C |
| Sample temperature | 37°C |
| Injection volume | 0.5 µl |

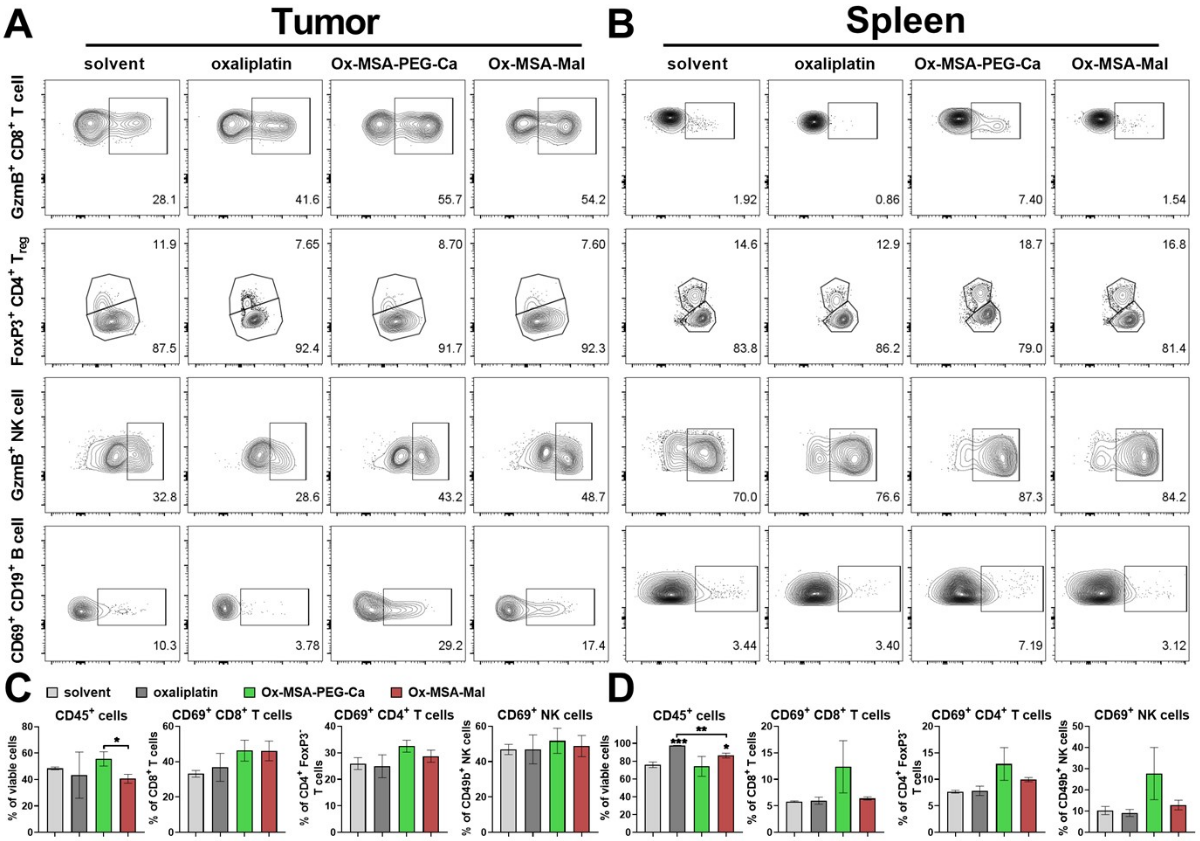


**Figure S12.** Immune cell activation and tumor infiltration following treatment with MSA-2-releasing platinum(IV) prodrugs as compared to free oxaliplatin. CT-26 colorectal cancer-bearing BALB/c mice (n=4/group) were dosed twice (day 1 and 4) with the indicated drugs in all cases equimolar to 9 mg/kg oxaliplatin. 24 h after the second dose animals were euthanized. Immune cell populations were quantified in tumor (A, C) and spleen tissues (B, D) by flow cytometry. (A, B) Representative gates of the immune cell populations identified in Figure 8 of the main text are depicted. (C, D) Frequency of the indicated immune cell populations (as percentage of CD45+ cells) and their activation state (as percentage of respective immune cell compartment) are depicted as mean ± SEM. The gating strategy used to identify the indicated immune cell populations is depicted in Figure S14. The statistical significance between the groups was tested by Student's t-test. *$p < 0.05$; **$p < 0.01$; ***$p < 0.001$.

**UPDATED 13 May 2025. The original version of this SI was first published on 04 April 2025. This version of the SI replaces the previous version in which Figure S3 was not shown correctly.**

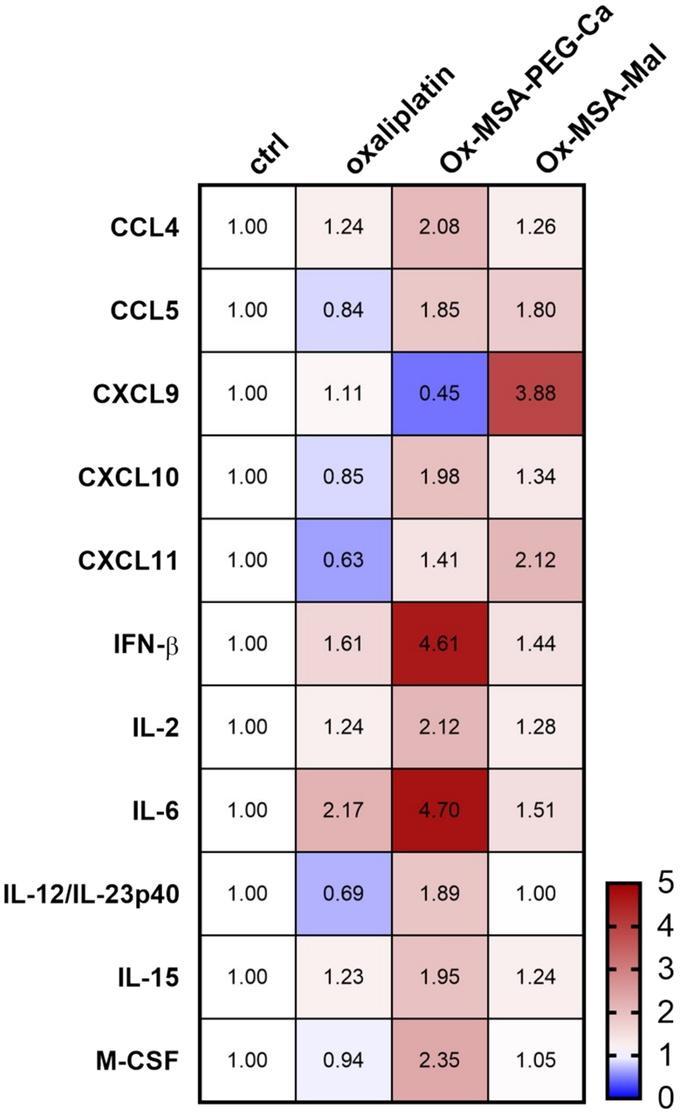


**Figure S13.** Cytokine and chemokine secretion in the plasma of animals following exposure to MSA-2-releasing platinum(IV) prodrugs or reference compounds. CT-26 colorectal cancer-bearing BALB/c mice (n=4/group) were dosed twice (day 1 and 4) with the indicated drugs in all cases equimolar to 9 mg/kg oxaliplatin. 24 h after the second dose animals were euthanized. Immediately before euthanization blood was drawn from the submandibular vein, plasma was isolated and analyzed for secreted cytokines and chemokines by a Luminex detection platform. Depicted mean cytokine/chemokine levels are normalized to solvent-treated animals (ctrl). ctrl = control.

**UPDATED 13 May 2025. The original version of this SI was first published on 04 April 2025. This version of the SI replaces the previous version in which Figure S3 was not shown correctly.**

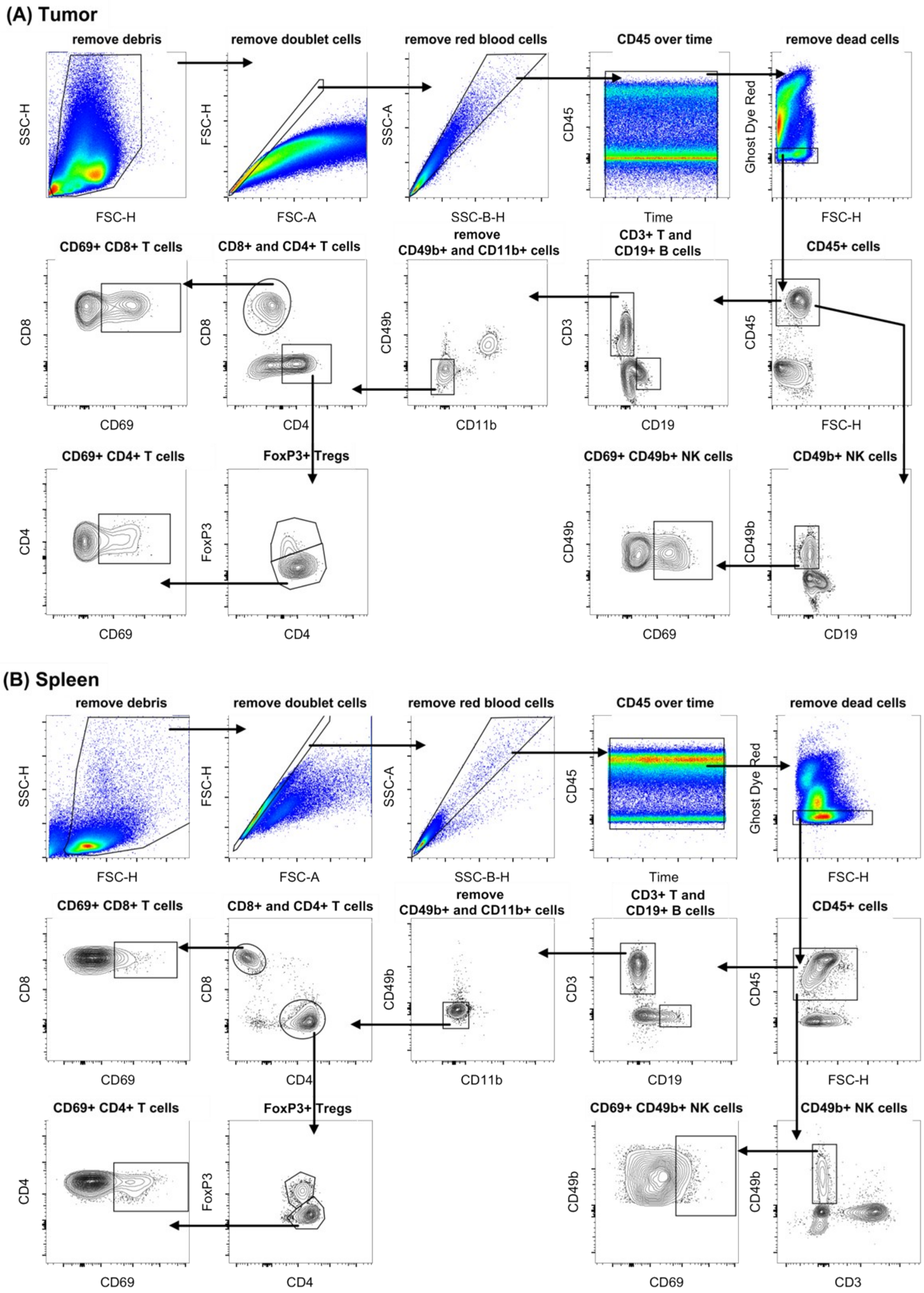


**Figure S14.** Gating strategy used for the identification of immune cell populations. Immune cell populations were identified in tumor (A) and spleen tissues (B) by flow cytometry. One representative sample is depicted.

**UPDATED 13 May 2025. The original version of this SI was first published on 04 April 2025. This version of the SI replaces the previous version in which Figure S3 was not shown correctly.**

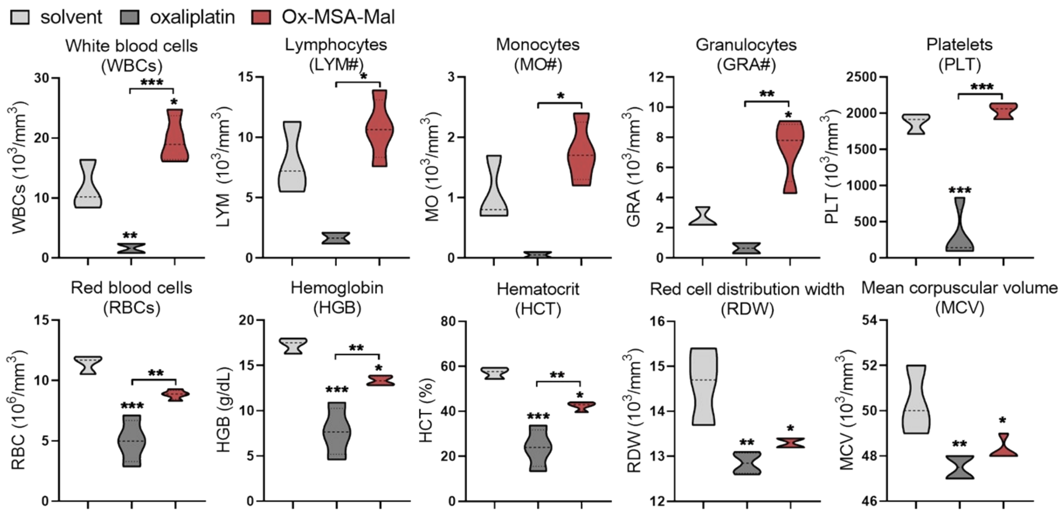


**Figure S15.** Long-term impact of the investigated drugs on hematological parameters. Peripheral blood was drawn from the submandibular vein of C57BL/6 mice 72 h after the fourth dose. Hematological parameters (WBC, white blood cells; LYM, lymphocytes; MON, monocytes; GRA, granulocytes; PLT, platelets; RBC, red blood cells; HGB, hemoglobin; HCT, hematocrit; RDW, red cell distribution width; MCV, mean corpuscular volume) were assessed using a Vet ABC hematology analyzer. Data are depicted as violin plots (the median and quartiles are shown by bold and dotted lines, respectively). The statistical significance of differences was tested via one-way ANOVA (with the Tukey multiple comparison test). * p < 0.05; ** p < 0.01; *** p < 0.001.

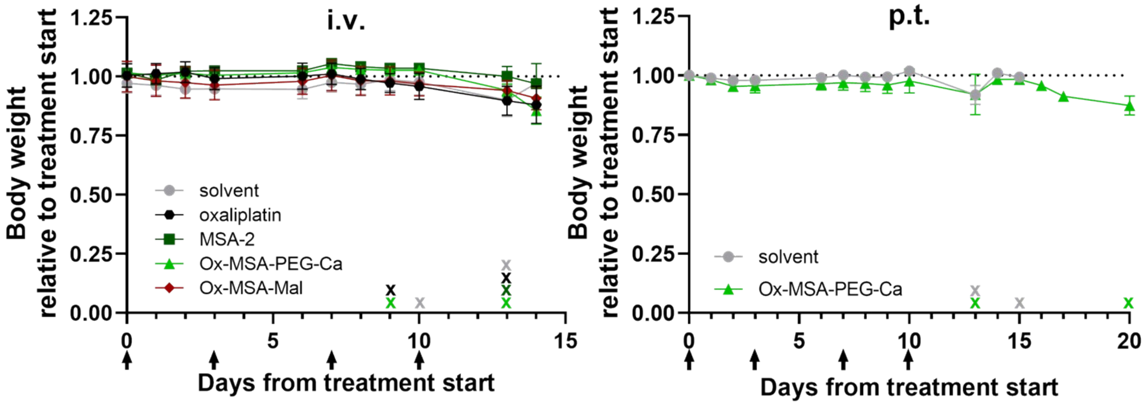


**Figure S16.** Body weights of animals treated with platinum(IV) complexes and impact of the application route. CT-26 colorectal cancer-bearing BALB/c mice (n=4/group) were dosed (A) intravenously (i.v.) or (B) peritumorally (p.t.) twice-weekly for two weeks with the indicated drugs in all cases equimolar to 9 mg/kg oxaliplatin. Euthanasia of individual animals is indicated by an X symbol along the x-axis. Data points are depicted as mean ± SEM. p.t. = peritumoral.

**UPDATED 13 May 2025. The original version of this SI was first published on 04 April 2025. This version of the SI replaces the previous version in which Figure S3 was not shown correctly.**

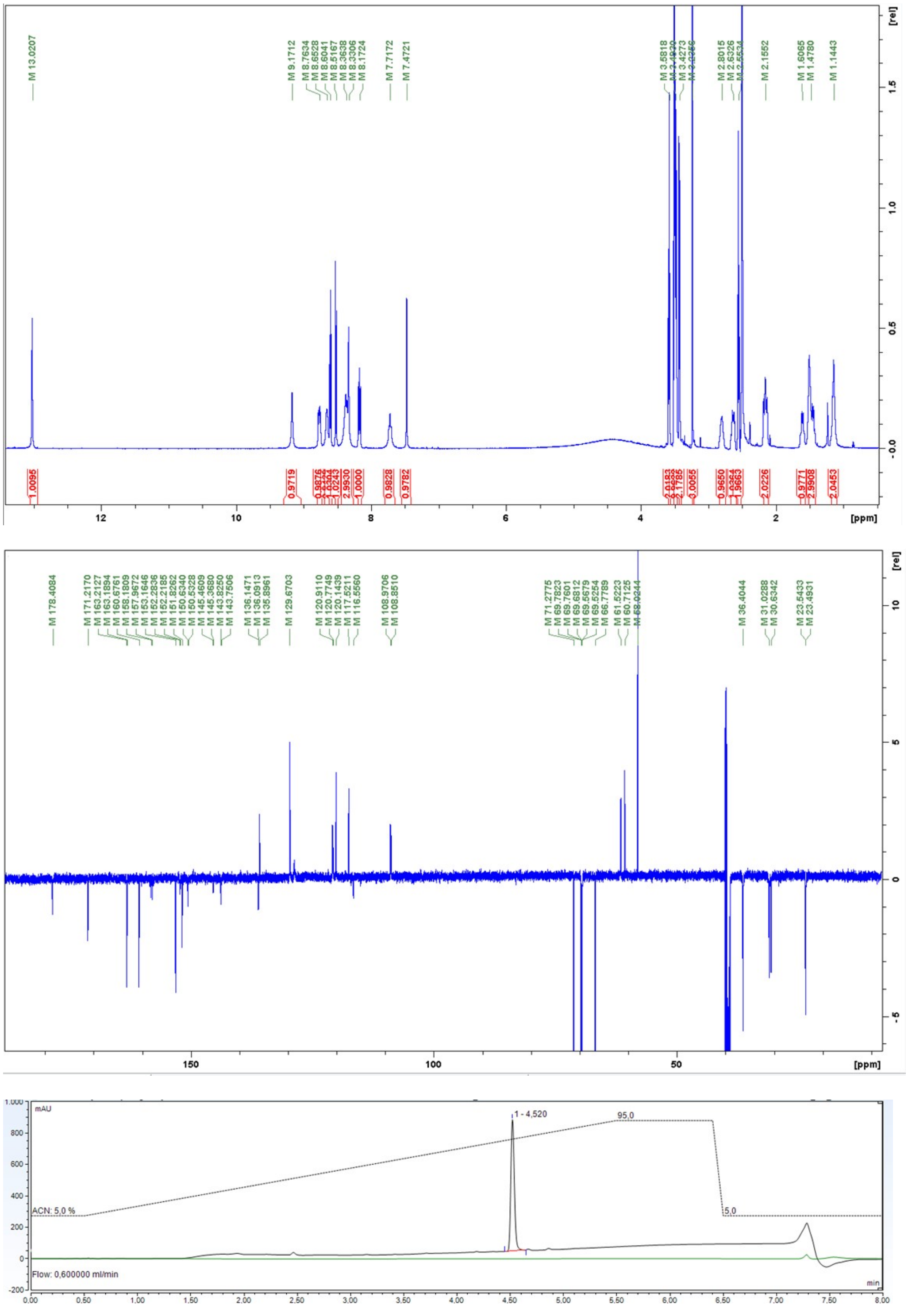


**Figure S17.** $^{1}H$ and $^{13}C$-NMR spectra of **Ox-SR-PEG-Es**, measured in DMSO-d6, and a UHPLC chromatogram (220 nm), measured with MeCN (+0.1% HCOOH) and $H_2O$ (+0.1% HCOOH) using a 5-95% gradient.

**UPDATED 13 May 2025. The original version of this SI was first published on 04 April 2025. This version of the SI replaces the previous version in which Figure S3 was not shown correctly.**

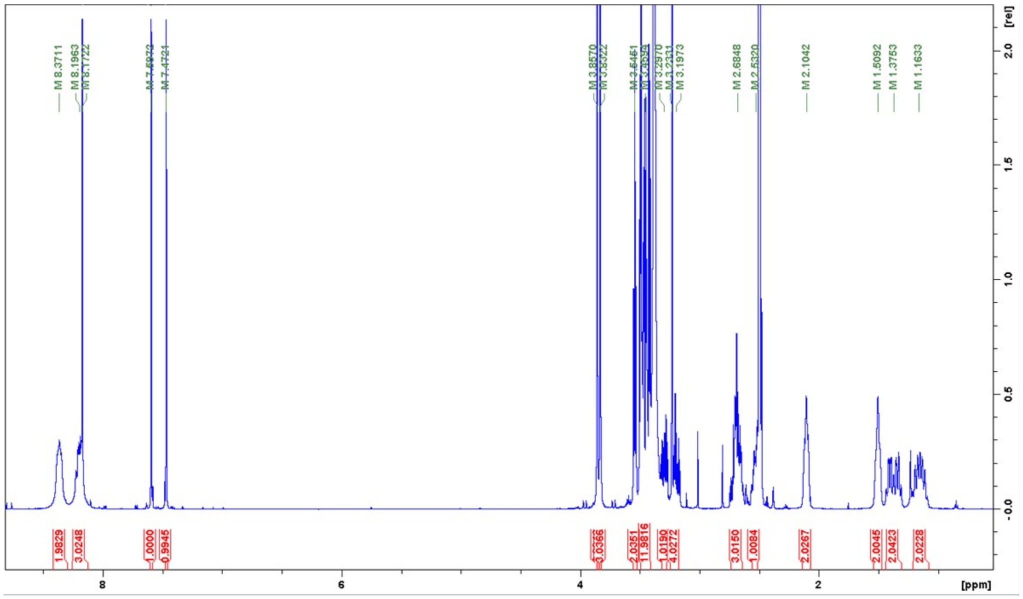


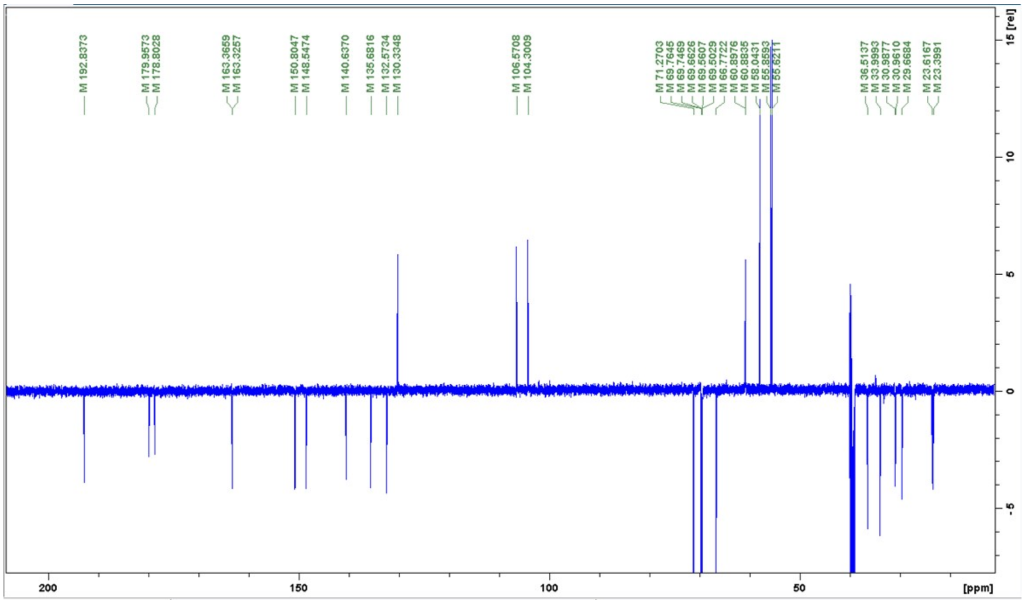


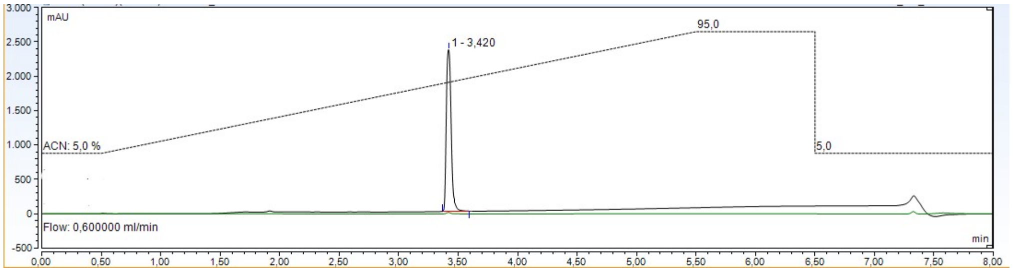


**Figure S18.** $^1$H and $^{13}$C-NMR spectra of **Ox-MSA-PEG-Es**, measured in DMSO-d6, and a UHPLC chromatogram (220 nm), measured with MeCN (+0.1% HCOOH) and $H_2O$ (+0.1% HCOOH) using a 5-95% gradient.

**UPDATED 13 May 2025. The original version of this SI was first published on 04 April 2025. This version of the SI replaces the previous version in which Figure S3 was not shown correctly.**

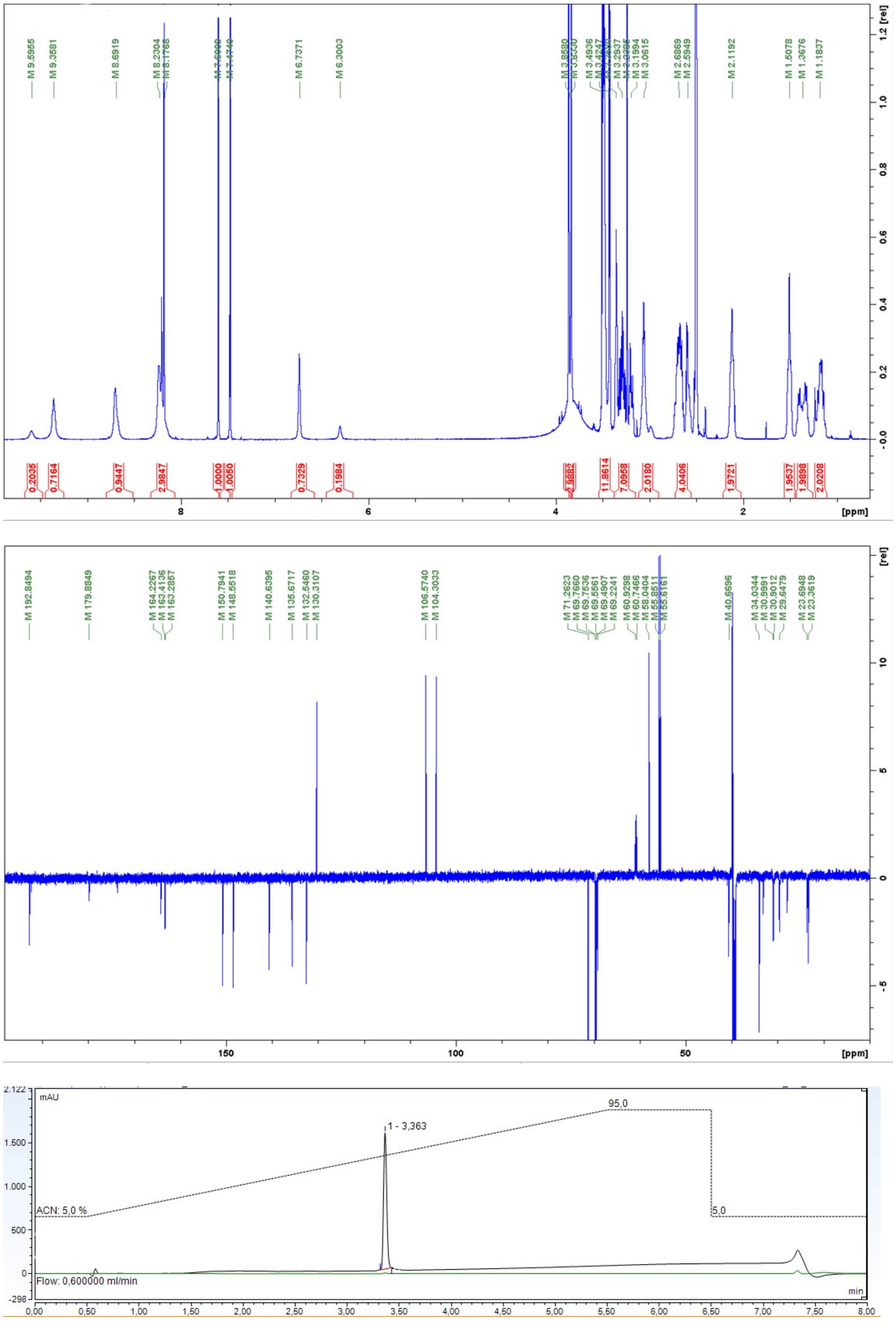


**Figure S19.** $^{1}$H and $^{13}$C-NMR spectra of **Ox-MSA-PEG-Ca**, measured in DMSO-d6, and a UHPLC chromatogram (220 nm), measured with MeCN (+0.1% HCOOH) and $H_2O$ (+0.1% HCOOH) using a 5-95% gradient.

**UPDATED 13 May 2025. The original version of this SI was first published on 04 April 2025. This version of the SI replaces the previous version in which Figure S3 was not shown correctly.**

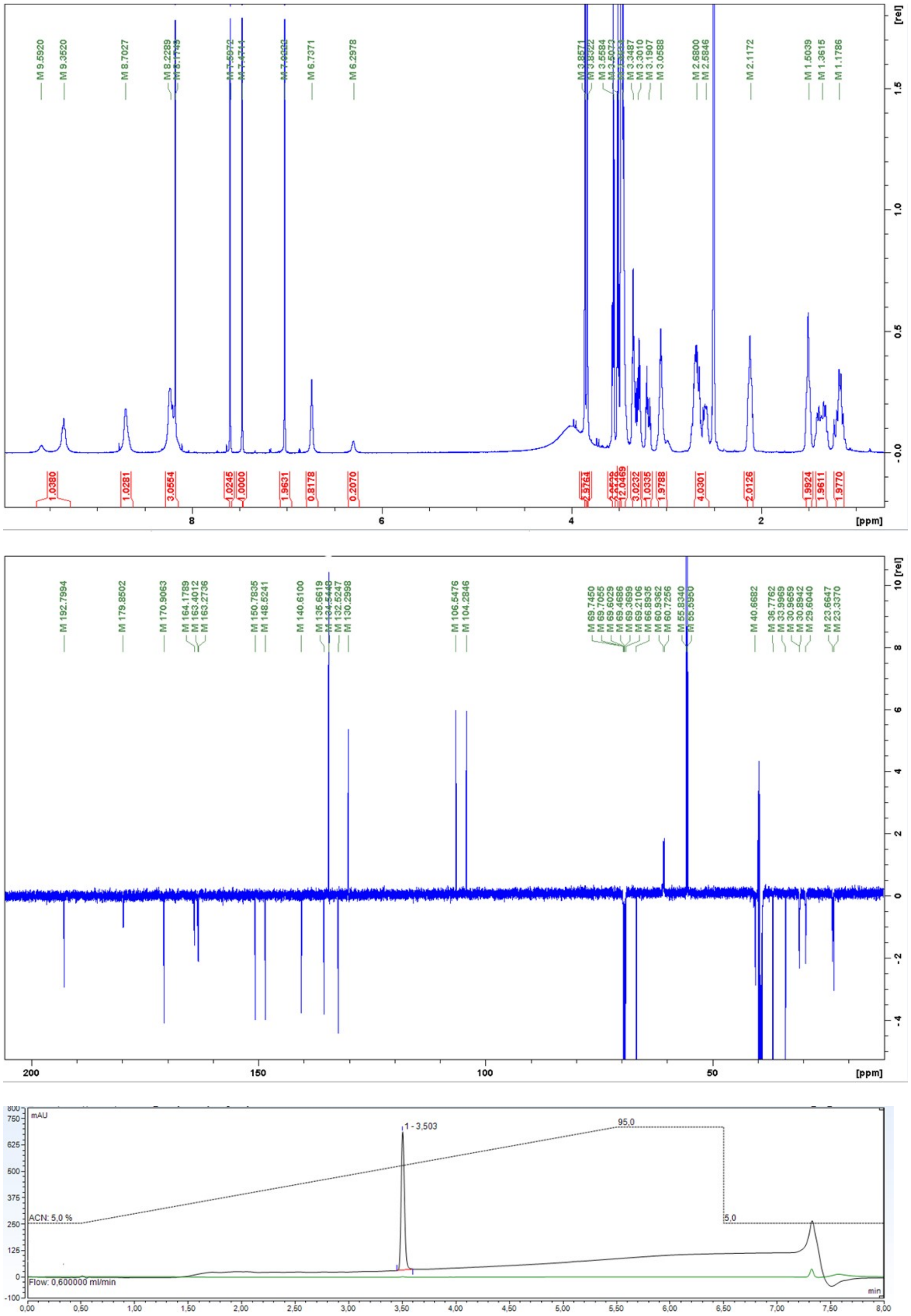


**Figure S20.** $^{1}$H and $^{13}$C-NMR spectra of **Ox-MSA-Mal**, measured in DMSO-d6, and a UHPLC chromatogram (220 nm), measured with MeCN (+0.1% HCOOH) and $H_2O$ (+0.1% HCOOH) using a 5-95% gradient.
